# Endocrine Resistance in Hormone Receptor Positive Breast Cancer–From Mechanism to Therapy

**DOI:** 10.3389/fendo.2019.00245

**Published:** 2019-05-24

**Authors:** Aradhana Rani, Justin Stebbing, Georgios Giamas, John Murphy

**Affiliations:** ^1^School of Life Sciences, University of Westminster, London, United Kingdom; ^2^Department of Surgery and Cancer, Imperial College London, London, United Kingdom; ^3^Department of Biochemistry and Biomedicine, School of Life Sciences, University of Sussex, Brighton, United Kingdom

**Keywords:** endocrine resistance, breast cancer, signaling, estrogen (E2), estrogen receptor

## Abstract

The importance and role of the estrogen receptor (ER) pathway has been well-documented in both breast cancer (BC) development and progression. The treatment of choice in women with metastatic breast cancer (MBC) is classically divided into a variety of endocrine therapies, 3 of the most common being: selective estrogen receptor modulators (SERM), aromatase inhibitors (AI) and selective estrogen receptor down-regulators (SERD). In a proportion of patients, resistance develops to endocrine therapy due to a sophisticated and at times redundant interference, at the molecular level between the ER and growth factor. The progression to endocrine resistance is considered to be a gradual, step-wise process. Several mechanisms have been proposed but thus far none of them can be defined as the complete explanation behind the phenomenon of endocrine resistance. Although multiple cellular, molecular and immune mechanisms have been and are being extensively studied, their individual roles are often poorly understood. In this review, we summarize current progress in our understanding of ER biology and the molecular mechanisms that predispose and determine endocrine resistance in breast cancer patients.

## Introduction

The complex association of endocrine ablation and breast cancer (BC) was discovered in 1896 when Beatson revealed that oophorectomy on an advanced cancer patient led to a pronounced and marked response (1). The estrogen receptor (ER) was first discovered in 1958 by EV Jensen and subsequent studies showed that estrogen (E2) is implicated in BC pathogenesis and nurtures the surge in ER expressing BC cells (2, 3). E2 modulates its activity through its two ERs: ERα (ERα) and β (ERβ) (4, 5). *ER*α was cloned in 1985 by Pierre Chambon's group and *ER*β was cloned in 1996 by Jan-Ake Gustafsson's group (6, 7).

However, it is the *ER*α which is expressed predominantly in breast tumors, and considered the most suitable target for hormonal therapy (8). Consequently, most treatments are directed at reducing the levels of E2 or inhibiting the E2-mediated signaling. Endocrine therapy has been prevalent ever since the discovery of endocrine cancers and it is, to date, one of the most effective treatments in ER-positive (ER+) BC. At least six distinct therapeutic modalities dictate endocrine therapy, namely the selective ER modulators (SERMs), the selective ER down-regulators (SERDs), aromatase inhibitors (AIs), mTORC1 inhibitors in combination with aromatase inhibitors, and cyclin dependent kinases 4 and 6 (CDK4 and CDK6) inhibitors in combination with AIs and CDK4/CDK6 inhibitors in combination with SERDs (9).

Tamoxifen, a SERM that acts by blocking the ER, has been ubiquitously used over the last four decades as the treatment of choice in pre-menopausal BC patients, although in the adjuvant setting, it was the drug of choice in post-menopausal women with HR+ BC (10, 11). AIs have been shown to be effective in post-menopausal women and might be effective in patients resistant to SERMs (12–14). With the discovery of endocrine therapies, the population of BC cells have been evolving too and overcoming endocrine resistance is a major cause of concern.

ER signaling is a complex cascade of events and the function of ER activity is dependent on the microenvironment within the cell at the molecular level, i.e., other signaling molecules, coactivators, corepressors, and genomic sequence of consensus binding sites. These are some of the determinants leading to the inefficiency of endocrine therapy and are associated with integral or assumed endocrine resistance. The cellular and molecular milieu can determine the transformation from an E2-dependent to E2-independent cluster of proteins, which subsequently go on to regulate genes in the absence of the E2-ER signaling.

In the Immediate Preoperative Anastrozole, Tamoxifen, or Combined with Tamoxifen (IMPACT) neoadjuvant trial, BC cell proliferation was measured by Ki67 immunohistochemistry (IHC) and it was demonstrated that almost 40% of patients presented with a high correlation of a 5-years recurrence free survival (RFS) upon short term anti-E2 therapy (15). In this study, the AI anastrozole was more effective in suppressing Ki67 expression. The larger adjuvant Arimidex, Tamoxifen Alone or in Combination (ATAC) and Breast International Group (BIG) trials also reported that the set of patients exhibiting significant inhibition of cell proliferation upon endocrine therapy had a larger number of tumor cells that were hormone sensitive/dependent (11, 16). Whole exome sequencing of operable ER+ patient tumors after estrogen deprivation by an AI, has the potential to identify useful biomarkers and suitable therapy in a subset of BC patients (17). Large genomic datasets have been generated from ER+ BC patients revealing a catalog of somatic mutations within genes and each of these datasets have provided a wealth of information (18, 19). A recent study identified association with clinical variables and *MAP3K1* and *TP53* mutations were predictors of good and poor response, respectively (19). These findings were in line with observations in the METABRIC dataset (20).

An in-depth characterization of tumors through large integrated genomic landscape studies on metastatic breast cancer (MBC) patients has provided valuable insights into a few of the genomic drivers, the role of heterogenic genomic architecture of cells within the tumor, the cellular and molecular determinants that define response to endocrine therapy along with identified novel biomarkers and therapies (9, 21, 22). These studies have demonstrated a central clonal hub at the primary tumor site and acquired mutations and drivers that promotes metastasis (21). One such study identified the SWI-SNF and JAK2-STAT3 pathways as potential therapeutic targets (21). Another of the recent studies identified at least four separate clusters of cells: 1. A cluster of tumorous cells possessing *ER*α mutations 2. A cluster of cells harboring mutations within the RAS/RAF/MEK/MAPK pathway 3. A cluster of cells with mutations in a variety of transcriptional factors and 4. A cluster of cells with unknown mechanisms (9). Although *ER*α mutations are common in a small proportion of endocrine resistant BC patients, the mechanistic details of endocrine resistance in the remaining patients remains elusive and has been discussed in further details below. Clinico-genomic factors as well as the role of the immune system and its associated tumor microenvironment (TME) provide a strong rationale for stratifying therapeutic approaches in endocrine resistant BC patients.

The immune system, both innate and adaptive play a pivotal role in BC. The microenvironment and the soluble mediators involved, including cytokines, dictate to a large extent, the ability of immune cells to either subdue the proliferating cancer cells or support the growth and metastatic capacity of BC cells. Lymphocyte infiltration has been considered in a recent study and this has reinforced the importance of the immune system in ER+ BC (23). While the authors did not observe any prognostic significance on the scores for immune cell abundance accounting for tumor infiltrating lymphocytes (TILs) in histology sections, they did observe prognostic significance for the immune scores based on spatial heterogeneity of the TILs and an increase in spatial clustering across late recurrent ER+ cancer.

**Figure 1:**
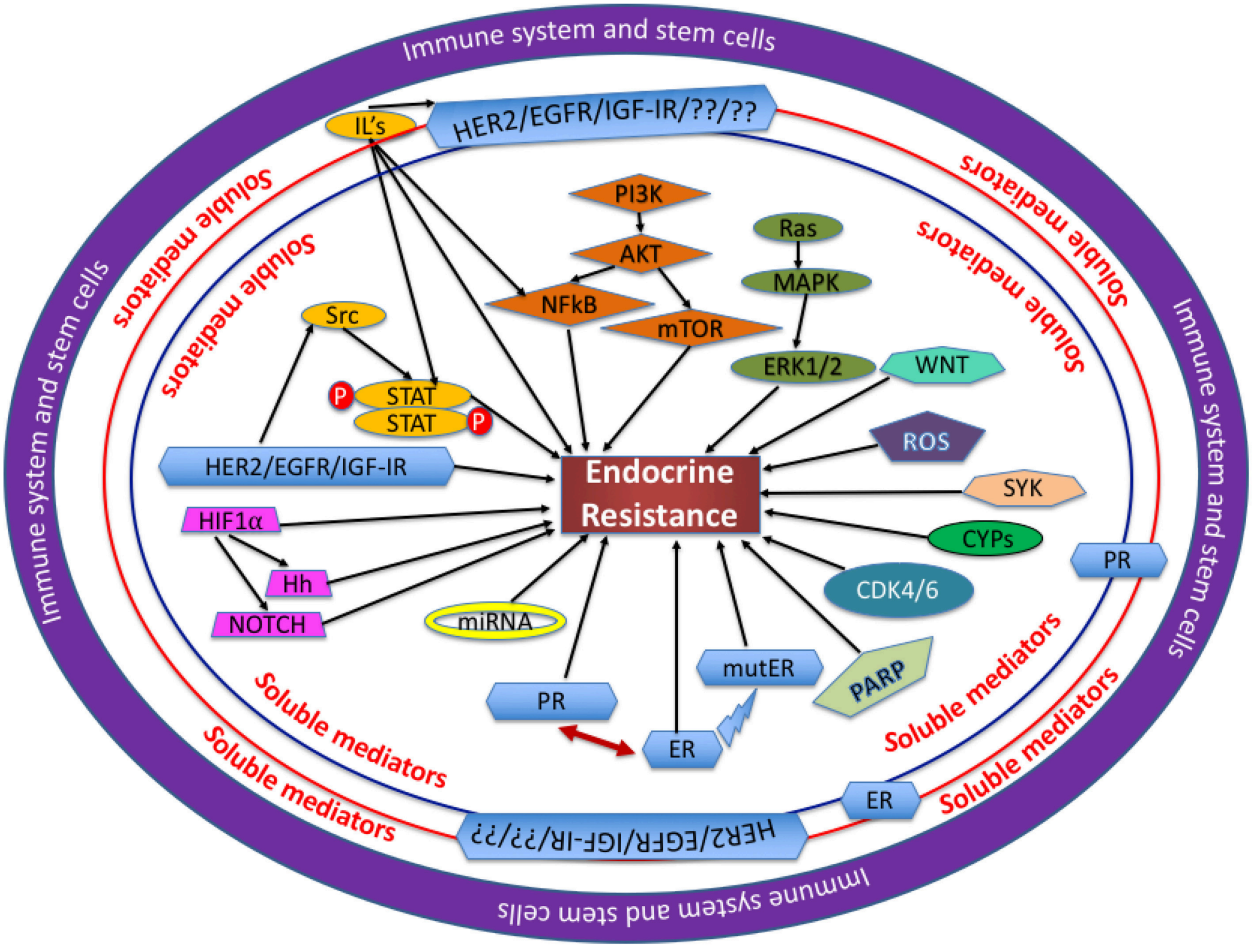
Mechanisms involved in endocrine resistance: Several proteins, soluble mediators and transcription factors are assimilated and function cohesively in a complex network, with each entity playing a unique role through the regulation of its own cascade of events as mentioned in each section of this review. In the opinion of the authors, the immune system and the stem cells are at the center of dysregulation observed in the proteins and pathways involved. The soluble mediators, like hormones, cytokines, and chemokines all play a crucial role in BC cells becoming endocrine resistant. IL, interleukin; ROS, reactive oxygen species; mutER, mutations in ERα.

A thorough study of the molecular structure, components and cascade of events leading to endocrine resistance will highlight suitable schemes and strategies for therapeutic targeting. Illustrated in Figure 1 are the various factors leading endocrine sensitive cells toward reduced endocrine sensitivity/endocrine resistance. This review discusses in detail the various intricate and sophisticated web of pathways involved in ER signaling and highlights schemes for treatment.

## 1. Estrogen Receptor and Signaling

ER is a nuclear receptor and belongs to the steroid-thyroid-retinoid receptor superfamily (6). The human ER is comprised of two subdivisions: ERα and ERβ, located on chromosomes 6 and 14 at 6q21.5 and 14q23.2, respectively (7, 24). In some tissues, like the uterus, mammary gland, testis, pituitary, liver, kidney, heart, and skeletal muscle, *ER*α appears to dominate, whereas in the ovary and prostate, *ER*β is highly expressed. The role of ERα in breast malignancy is well-documented, while the function of ERβ remains ambiguous (8). It is understood that ERβ has contrasting effects to ERα, and inhibits the stimulatory effects of E2 on cell proliferation. Studies have shown that downregulation of *ER*β contributes to tumor progression and the chances of survival increase with an increase in the expression of *ER*β (25, 26).

**Figure 2:**
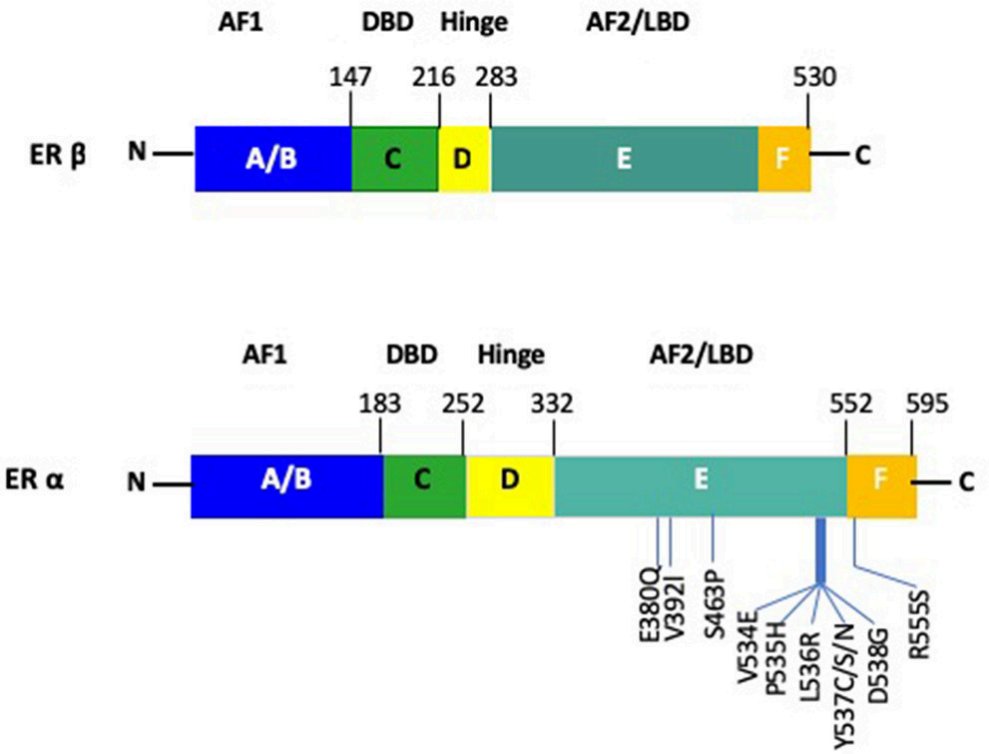
Schematic figure of the structure of ERα and ERβ. The AF1 site is located at the N-terminus A/B domain. The DBD and dimerization site is present within the C domain. The nuclear localization signal is contained in the D domain. The E/F domain contains the AF2 site as well as the ligand binding domain along with a dimerization site. Notable mutations within ERα are depicted for the ERα gene.

Both receptor subtypes share similarity at the protein level and are comprised of domains denoted as A to F (Figure 2) (27, 28). However, there is only limited homology between the two receptors, with 95% in the DNA binding domain (DBD), 50% in the ligand binding domain (LBD) and 15% in the N terminal domain of the two receptors (29). The ERα and ERβ contains two transactivation domains, the activation function 1 (AF1) and activation function 2 (AF2), with the N-terminal AB domain (containing the AF1), responsible for constitutive ER activity (30). The DNA-binding domain (DBD) or C domain, binds to the palindromic sequence, GGTCAnnnTGACC and leads to dimerization of the ER (28). The D domain/hinge domain leads to nuclear transport, while the E domain/Ligand-Binding Domain (LBD), is responsible for ligand binding (31–33). Located at the carboxy terminus is the F domain/AF2, which is responsible for E2 dependent activation of the ER. This region modulates the functions of the AF1 and AF2 domains (34). Consistent with previous reports on the role of polymorphisms in the *ER*α and *ER*β genes on endocrine resistance, recent large scale projects, like The Cancer Genome Atlas (TCGA), have indicated similar results, in that *ER*α mutations were present in only 0.5% of BC patient samples and *ER*α amplification in 2.6% (35).

Upon binding of E2 to ER, a series of successive triggers, results in the translocation of chaperone proteins from the ERα, receptor dimerization, phosphorylation and subsequent binding of ER as a dimer to consensus binding sites on the DNA, known as E2 response elements (EREs). The EREs are located in the 5′ untranslated region (UTR) of genes, although it is well-known that ER can bind to intronic as well as distal regions of the transcription start site of a gene and that ERα binds within the enhancer rather than the promoter regions and also facilitates transcription through long distance interactions by DNA looping (36–38). ER signaling can be triggered by nuclear and non-nuclear mechanisms. At the molecular level, ER regulates the expression of genes by both canonical and non-canonical pathways, also known as “nuclear-initiated steroid signaling (NISS)” pathways (15). The consensus palindromic ERE was initially defined as 5′-GGTCAnnnTGACC-3′, from the *X. laevis* vitellogenin gene, although the ERE in humans ranges from 3 to 5 nucleotides between the penta half sites (GGTCA(n)_3−5_TGACC (39, 40). When ER binds to the ERE on the DNA, it leads to gene transcription of target genes, regulated by synergistic activity of AF2 and AF1. Additional co-activator (Co-A), specificity protein 1 (SP1) and activator protein 1 (AP1) are recruited to the ER/DNA complex and can regulate cellular function by upregulating or downregulating gene transcription (41, 42) (Figure 2). Essentially, the activity of the ER is modulated by post-translational modifications which include, phosphory/acety/palmitoy/sumoy-lations and ubiquitination (Table 1). ERα is phosphorylated at Ser118, 104, 106, and Tyr537, acetylated at Lys266, 268, 299, 302, and 303, palmitoylated at Cys447, sumoylated at Lys 299, 302, and 303 and ubiquitinated at Leu 429 and Ala 430 among few others. In the last decade, studies have shown that a proportion of target genes are regulated using a more complex machinery, where more than one ERE-consensus sequence and/or non-consensus ERE sites are present in the promoter region (47).

**Table 1:** Post-translational modifications in ERα.

**Site of modification**	**Modification**	**Protein association**	**Function**
Ser46/47	Phosphorylation	PKC	Activates: transcription
Tyr52	Phosphorylation	c-Abl	Activates: protein stability, transcription, cell growth/invasion
Ser102	Phosphorylation	GSK3	Activates: transcription
Ser104/106	Phosphorylation	GSK3, Cyclin A-Cdk2, MAPK/ERK	Activates: transcription, coactivator binding
Ser118	Phosphorylation	MAPK/ERK, Cdk7, GSK3, IKKα, ILK, EGFR, IFG-IR, DNA-PK, RET	Down-regulates: transcription, Activates: RNA splicing, Dimerization, transcription, coactivator binding, protein stability, cell growth/invasion
Ser154	Phosphorylation	AKT	Unknown
Ser167	Phosphorylation	AKT, p90 RSK, S6K1, IKKα, CK2, RET	Down-regulation: transcription, Activates: transcription, DNA binding, stability
Ser212	Phosphorylation		Activates: DNA binding, transcription
Tyr219	Phosphorylation	c-Abl	Activates: dimerization, DNA binding, protein stability, transcription, cell growth/invasion
Ser236	Phosphorylation	PKA	Activates: dimerization, transcription, DNA binding
Arg260	Methylation	PRMT1	Non-genomic signaling
Lys266	Acetylation Sumoylation	p300, SUMO-1	Activates: DNA binding, transcription
Lys268	Acetylation Sumoylation	p300, SUMO-1	Activates: DNA binding, transcription
Ser282	Phosphorylation	CK2	Activates: transcription Inhibits transcription
Ser294	Phosphorylation	Proline directed kinase	Activates: transcription
Lys299	Acetylation Sumoylation	p300, SUMO-1, Ubiquitin, SET7	Inhibits transcription Activates: DNA binding, transcription
Lys302	Acetylation Methylation Ubiquitylation Sumoylation	p300, SUMO-1, Ubiquitin, SET7	Inhibits transcription Activates: DNA binding, transcription, Proteasomal degradation
Lys303	Acetylation Ubiquitylation Sumoylation	p300, SUMO-1, Ubiquitin	Inhibits transcription Activates: DNA binding, transcription, Proteasomal degradation
Ser305	Phosphorylation	PAK1, PKA, Akt	Activates: transcription, DNA binding, coactivator binding, cell growth/invasion
Thr311	Phosphorylation	p38-MAPK	Activates: nuclear/subcellular localization, transcription, coactivator binding
Leu429	Ubiquitylation		Activates: transcription Inhibits transcription
Ala 430	Ubiquitylation		Activates: transcription Inhibits transcription
Cys447	Palmitoylation	PAT	Plasma membrane localization
Tyr537	Phosphorylation	Src, EGFR	Activates: E2 binding, dimerization, DNA binding, transcription, coactivator binding, Proliferation
Ser554	Phosphorylation		
Ser559	Phosphorylation	CK2	Activates: transcription Inhibits transcription

*Adapted from Murphy et al. and Le Romancer et al. (43–46)*.

Both the AF1 and AF2 domains are crucial, but the AF2 activation serves as the binding region for coactivators and corepressors (48, 49). The predominant steroid receptor coactivator (SRC) are ERAP-160, RIP-140, SRC-1, CBP, p300, TIF2, and AIB1 (49), while the corepressor is SMRT (50). A recent study identified Oct4 as a cofactor associated with ERα to promote tamoxifen resistance (51). In this study, enhanced expression of Oct4 led to a tamoxifen dependent increase in the proliferative potential of the tumor. This is important in the context of endocrine resistance, since the array of molecules mobilized by the ER is dependent on the organization of the E2 mediated ER-ERE complex (52).

**Figure 3:**
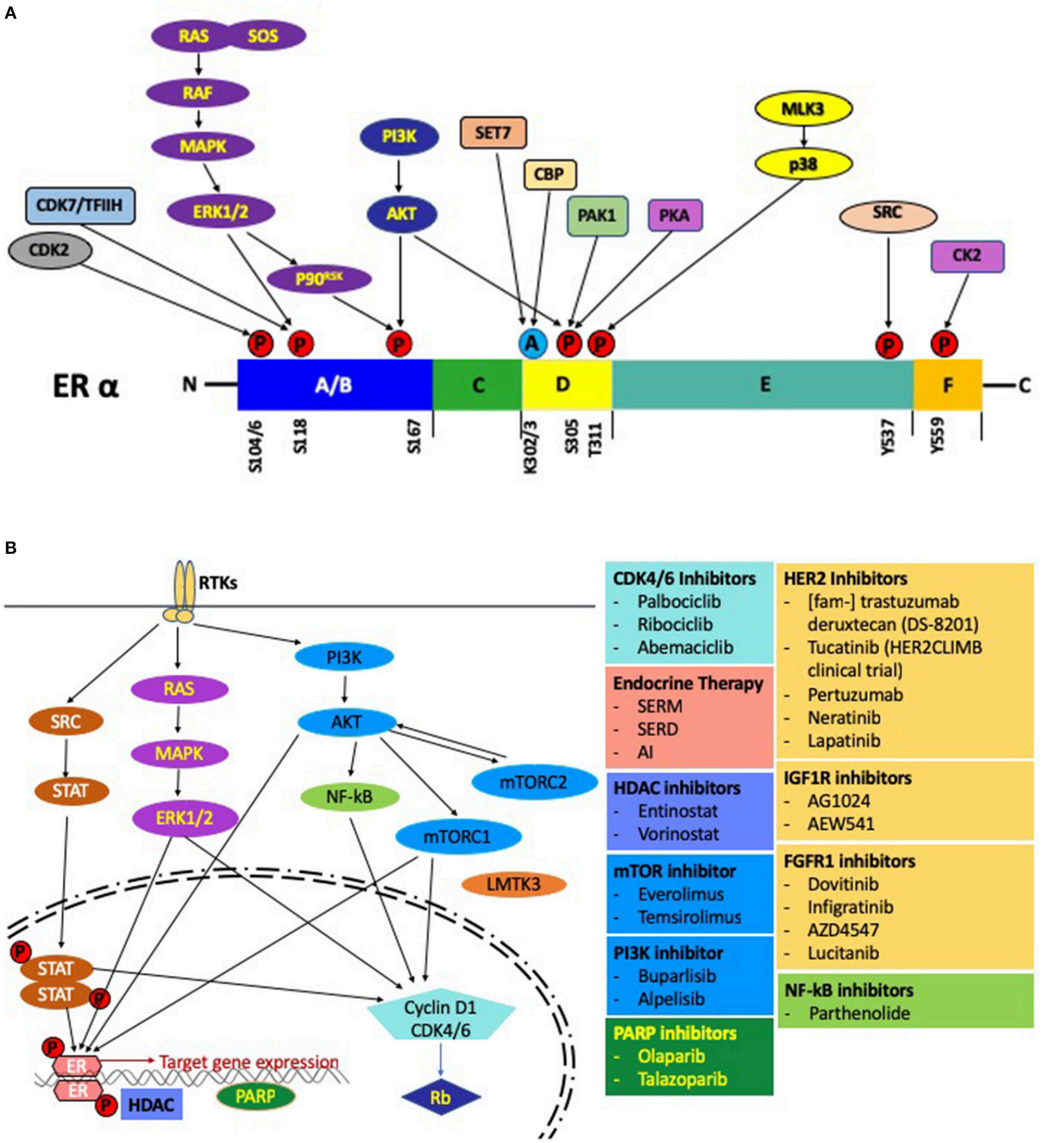
**(A)** Post-translational modifications of the ER. Activation of the growth factor receptor tyrosine kinases leads to phosphorylation of the ER through the RAS-MAPK and PI3K-AKT pathways. Several other pathways, including the CDK2 complex, CDK7/TFIIH complex can also phosphorylate the ER. Sensitivity to E2 is modulated by acetylation of the ER by src (CREB-binding protein). Pictorially represented above are the domains of the ER with phosphorylation/acetylation sites and the protein kinases mediating these modifications. **(B)** Regulation by E2 at the molecular level: A schematic representation of the pathways and the associated small molecule inhibitors involved in endocrine resistance.

In contrast, the AF1 domain is not dependent on a ligand and regulates gene transcription even in an ERα deletion mutant (53). In the ligand independent mechanism, activation of AF1 by phosphorylated ER at Ser^104^, Ser^106^, Ser^118^, Ser^167^, and Ser^305^ is mediated by crosslink and crosstalk between MAPK, PKA, PI3K/AKT and cyclin-dependent protein kinase 2/7 (Cdk2/7) pathways (54–58) (Figure 3A). The two prominent pathways mobilizing this cascade are those of the EGFR and IGFR1 (59). In response to E2, activation of AF1 leads to a concomitant action on the AF2 assisted by a complex of transcription factors to bring about a concerted effect on the ER mediated transcription (60, 61). These studies go on to highlight how redundancy can play a crucial role between signaling pathways and lead to an activation of the ER followed by subsequent activation, transcription and translation of ER regulated genes under conditions with inadequate E2 (62). This mechanism might lead to resistance against various endocrine therapies.

In the non-classical nuclear mechanism of ER signaling, the functions are mediated by activation of membrane bound ER, that occurs within minutes by activating protein kinase cascades. This rapid non-nuclear mechanism is also referred to as membrane-initiated steroid signaling (MISS) pathway (63). The responses are mediated via ER associated with caveolar rafts, which collaborate as dimers precisely with signaling adaptor proteins (64, 65).

E2 mediated responses are also conducted through a novel ER GPR30 (renamed GPER), a G protein-coupled receptor (66, 67). Since one in four ER+ patients do not respond to antioestrogens, GPER provides another possible mechanism of signaling, bypassing the E2-ER signaling mode (68, 69). Recent evidence suggests an association of GPER expression with resistance to endocrine therapy (70–72). GPER is unique in that it activates a whole new cascade of pathways distinct from those activated by the classical E2-ERα pathway (72). A fine crosstalk between GPER and HER-2 is considered to be an important pathway in the progression of breast cancer upon activation via E2 (73, 74). The GPER serves as an alternative E2 receptor since this pathway may be used by ER negative BC patients to drive HER-2 dependent proliferation (75). Thus, the interactions between classical ER and GPER is important to reveal the complex crosstalk and activation of non-nuclear mechanisms of E2 (76, 77).

Ligand-independent pathways are activated upon phosphorylation of ER by MAPK/ERK or PI3K/AKT (55, 78). Recent studies have shown a role for isoforms of ERα in the non-nuclear E2 signaling pathways (79, 80). The various pathways are an interplay of events and act in tandem. Thus, inhibition of one pathway or mode of E2 signaling does not completely abolish oestrogenic or ER regulatory actions. There is crosstalk between both nuclear and non-nuclear pathways and these effects are coactive and interdependent.

### 1a. Loss of ERα

Downregulation of ER is hypothesized as a cause of acquired resistance to tamoxifen, although it is not downregulated in approximately a quarter of tumors with acquired resistance to tamoxifen and a fifth of the tamoxifen resistant cancers will go on to be responsive to treatment with an AI or a SERM (e.g., Fulvestrant) (81, 82). Endocrine therapy is effective in ER + BCs where they are dependent on ER activation to exert their effects on growth and differentiation. Thus, it is the expression of ER, which determines response to endocrine therapy, and a lack of the ER is the principal cause of de novo resistance to endocrine therapy.

Cyclical and continuous methylation/demethylation of CpG dinucleotides is a prominent characteristic of ER and its target genes (83). A small proportion of BCs presenting with non-existent *ER*α gene expression have an intrinsic gain in CpG site methylation (84, 85). Another theory suggests that an increase in the deacetylation of histones, which limits transcription by condensing the nucleosome structure, could be a cause of non-existent ER transcription (85). Inhibition of the histone deacetylase, HDAC, revives ER transcription in BC cell lines which do not express the receptor (86). It is understood that a combination treatment with histone deacetylase inhibitor (HDACi) and DNA methyltransferase-1 (DNMT1) inhibitor will re-establish sensitivity to SERMs in BC cells not expressing the receptor (87). Thus, inhibitors to HDAC and DNMT1, by interfering with the epigenetic changes, could prove to be promising anticancer drugs in a small proportion of BCs presenting with a loss of the ER.

Epigenetic mechanisms play an important role in DNA methylation, chromatin modification and miRNA regulation. Recently, reports suggest that despite dysregulated signaling through the ER by SERMs and inadequate expression of the receptor, the target genes affected promote specific phenotypic changes in BC subtypes (88). Unsurprisingly, epigenetic mechanisms in the form of histone deacetylase inhibitors or demethylation agents are now being used in anti-cancer therapy (89–91). The ER and HDAC pathway crosstalk lead to changes in the activity and expression of ER and p21Waf1/Cip1, affecting cell proliferation, differentiation and survival (92). Hanahan and Weinberg have reviewed the importance of the loss of genomic methylation as well as hyper/hypo methylation of genes, which are involved in cell signaling, proliferation, or apoptosis and are thought to favor cancer progression (93).

Clinical studies have been done on endocrine resistant MBC patients using HDACi in combination with an endocrine agent. In one recent Phase II study done by Munster et al., an HDACi (vorinostat) was used in combination with tamoxifen or tamoxifen alone and it was noted that in the combination arm the objective response rate (ORR) was 19% and the clinical benefit rate (CBR) was 40%, thereby demonstrating that the combination is effective in overcoming endocrine resistance (94). Another Phase II study was done in patients treated with an AI, who had locally recurrent or ER+ metastatic breast cancer (MBC). Here, they were given either exemestane, an AI, with or without entinostat (a benzamide HDACi) and the combination arm again showed a benefit in terms of the progression-free survival (PFS) and overall survival when compared with the patients on exemestane alone (95) (Figure 3B). The HDAC inhibitor entinostat is being studied in BC patients on an AI (ClinicalTrials.gov Identifier: NCT02820961). Thus, HDACi's appear to be have an important role in reversing endocrine resistance.

### 1b. Mutated ERα

Mutations of the *ER* gene play a crucial role in the effectiveness of anti BC drugs. Although such mutations have not been detected in primary breast tumors, Fuqua et al. have detailed on the occurrence of an *ER*α variant with an A-to-G bp transition, which introduces a Lysine-to-Arginine substitution at residue 303 in almost a third of hyperplastic breast lesions (96). Although mutations in tamoxifen treated metastatic ER + BC patients present as *ER*α mutations, the majority are in the ERα LBD region, leading to constitutive activation of the ER (97–100). A point mutation, leading to a defective ER-ERE complex due to an alteration in the LBD was observed in tamoxifen resistant MCF7 cells (101, 102). A recent study identified hot spot mutations, like the Tyr537Ser, Tyr537Asn, and Asp538Gly (Y537S, Y537N, D538G) mutants within the LBD which favored the agonist structure of the ERα receptor, driving E2 independent transcriptional activity and proliferation of cancerous cells, leading to endocrine resistance (97). Whole genome RNA profiling in ER+/HER-2 negative BC patients revealed mutations/aberrations within the LBD domain of the *ER*α gene (17). The Y537S, D538G mutants had been previously identified and defined, while the V422del aberration is consistently observed in ER+ MBC (17). Thus, *ER*α mutations are more prevalent in MBC patients treated with AIs and occur in ~25–30% of these patients (103). The most prevalent mutations present in these patients are the D538G, Y537S, Y537N, Y537C, and E380Q. A recent study has indicated that constitutive transcriptional activity of the D538G mutation in *ER*, leads to overexpression which in turn leads to enhanced proliferation, thereby conferring resistance to tamoxifen (99).

Another point mutation in ER (nucleotide A908G), leads to an enhanced response upon activation by E2 and has been associated with invasive BCs (96). Such loss of regulation could contribute to the development of endocrine resistance as has been reported by Ellis et al., where they identified an *ER*α/*YAP1* fusion gene and defined its association with endocrine-resistant BCs (104). In the last few years several studies have been done to provide a complete set of mutations that could cause BC although in primary tumors, no mutation has been identified in the *ER* (97, 98, 105). In the metastatic scenario, several *ER* mutations have been identified by at least three other studies (97–99). Basically, several studies using next generation sequencing and liquid biopsies in cohorts of clinical trials since 2013, led to an interest in the high prevalence of *ER*α mutations in ER+ MBC patients with prior AI treatment (100, 106, 107). Mutations at the D538G and/or the Y537S, E380Q, Y537N, and Y537C sites on the *ER*α gene, have been studied as part of the BOLERO-2, SoFEA as well as the PALOMA-3 trial and a few others (106, 108). The results from these studies demonstrate the possible use of mutations at these Y537S, Y537N, Y537C, D538G, and E380Q sites on the *ER*α gene as marker to screen for endocrine therapy resistant BC (109). The Y537S and D538G mutant forms of *ER*α have distinct cistromes when compared to the E2-stimulated WT *ER* (*ER*α^*WT*^) form and have been demonstrated to drive endocrine resistance and metastasis (110). A recent study on a small molecule SERD, AZD9496, demonstrated that it binds to and downregulates the D538G/Y537S/Y537N/Y537C containing ERα proteins *in vitro*, leading to an appreciable inhibition in the rate of tumor progression (111). AZD9496 was more effective than fulvestrant in suppressing the growth of tumors driven by *ER*α^*WT*^ and *ER*α^*MUT*^ (Y537S) (112). The drug is also well-tolerated in ER+/HER2 negative advanced BC as demonstrated in a phase I clinical trial (113). Some recent SERDs are being developed to target ER in both their wild-type (*ER*α^*WT*^) and mutant forms (*ER*α^*MUT*^). The purpose of this study was to evaluate the efficacy of a novel orally bioavailable SERD, elacestrant (RAD1901), in preclinical models of ER+ BC. Elacestrant (RAD1901) is one such SERD that inhibits cell proliferation in ER+ BC cell lines and is being studied as part of clinical trials in ER+/HER-2 negative advanced BC where partial response as an effective SERD was demonstrated in heavily pre-treated ER+/ER mutant MBC and patients (ClinicalTrials.gov Identifier: NCT03778931 and NCT02338349) (114, 115). A structurally and chemically unique SERD, GDC-0927, induces tumor regression in ER+ MBC patients including those with *ER*α mutations (ClinicalTrials.gov Identifier: NCT02316509) (116, 117). Essentially, a range of SERDs are being developed with differential activities as ER antagonists to combat the clinical effectiveness of fulvestrant due to poor bioavailability. A recent study evaluated three recently developed ER ligands, GDC-0810, AZD9496 and GDC-0927 along with fulvestrant and it was GDC-0927 and fulvestrant that showed enhanced transcriptional suppression of the ER (118). A new class of drug, the selective estrogen receptor covalent antagonist (SERCA) with H3B-5942 being identified as an ERα antagonists that inactivates both the ERα^WT^ and ERα^MUT^ forms (119).

Thus, present studies to-date demonstrate that ER mutations are rare in primary tumors but appear to be reasonably frequent in the progression to endocrine resistance and can be used as biomarkers for prognosis/prediction of response to endocrine therapy along with promoting the development of therapeutic strategies. Evaluating and studying a variety of potent ER antagonists will promote the development of clinically effective SERDs (118).

## 2. Progesterone Receptor (PR) and Signaling

In BC, the Progesterone Receptor (PR) also plays an important role and its signaling has been at the center of various targeted therapies, including the selective progesterone receptor modulators (SPRM) (120). The PR is regulated by the ER and is required for mammary gland development. Progesterone binds to the PR, which is followed by receptor dimerization and translocation to the nucleus where it binds to the progesterone response elements (PRE) within the promoter/enhancer regions of PR target genes, thereby leading to either the upregulation or downregulation of those genes. Apart from binding to the PRE in its target genes, it also binds as a complex of transcription factors (PR in complex with AP1 and SP1) to regulate genes devoid of canonical PRE binding sites (121, 122). Indeed, a study of PR mediated regulation of oncogenic genes in PR regulated BC models has yielded information on its varied actions (123).

The PR exists as two isoforms, the PR-A and PR-B with the PR gene regulated by a complex of ERα-AP1-SP1 at an ERE motif half site on the PR gene (124, 125). Although normal mammary epithelial cells express separate receptors (ER and PR) on designated cells, the receptors are co-expressed in oncogenic cells (126). A dominant pathway takes over the functions of another during targeted therapy, in one such instance, a loss of the PR during endocrine therapy leads to the cancer becoming more aggressive. These patients have a poor survival outcome (127). Evidence from various studies, reflect the importance of the PR, in that, ER+ BC tumors presenting with PR-negative status have a poorer outcome than those with PR-positive (PR+) ones (128). A loss in PR leads to activation and upregulation of the PI3K pathway (129). A recent study demonstrated that the PR-B in a complex with ER and PELP1 promoted the regulation of E2 dependent ERα target genes associated with BC cell proliferation and tamoxifen resistance (130). In another study by Mohammed et al., PR was shown to be complexed with the known ERα co-factors, NRIP1, GATA3, and TLE3, upon stimulation with progesterone. They also concluded that activation of the PR led to the formation of an ERα-PR complex (131).

## 3. Receptor Tyrosine Kinases (RTK)

Receptor Tyrosine Kinases (RTK) are a family of receptors that are activated upon binding their respective ligands, which are mainly the growth factors, hormones or cytokines. The most prominent RTK's are the family of epidermal growth factor receptors (EGFR), Insulin-like growth factor-I receptor (IGF-IR), hepatocyte growth factor receptor (HGFR), vascular endothelial growth factor receptor (VEGFR), platelet-derived growth factor receptor (PDGFR), fibroblast growth factor receptor (FGFR), anaplastic lymphoma kinase (ALK), ROS protooncogene 1 (ROS1) and receptor like tyrosine kinase (RYK). An upregulation of RTKs is observed in breast cancer and is indicative of poor prognosis (132, 133). Upon binding to their respective ligands, the prominent pathways activated are the mitogen activated protein kinase (MAPK), janus kinase (JAK)/signal transducer and activator of transcription (STAT) and phosphoinositide (PI3K)/AKT pathways. A multitude of evidence suggests that inhibitors of RTK's can reverse therapeutic resistance in metastatic breast cancers (134) (Figure 3B).

### 3a. EGF/EGFR/HER2 Signaling

The ErbB family of growth factor receptors includes epidermal growth factor receptor (EGFR) (also called ErbB1/HER1, ErbB2/HER2, ErbB3/HER3, and ErbB4/HER4). Upon activation by ligands, such as EGF, which induces dimerization and autophosphorylation, signaling proceeds through various pathways including the MAPK-ERK and PI3K-AKT pathways (Figure 3B) (135). EGFR, HER2, HER3, and HER4 are all implicated to varying degrees in BC with their relative involvement in the order HER2-EGFR-HER3-HER4. HER2 expression is reported in 20–30% of BCs and HER3 is up-regulated in 10% of BCs, and its common association with HER2 makes its specific role difficult to determine (136).

With the ever increasing evidence for a role of ERα in signaling via EGFR, an association with DNA-synthesis has been observed in MCF7 cells, whereby an E2 antagonist led to a reduction in the phosphorylation of the EGFR, followed by reduced DNA synthesis and cytoskeleton formation through a Src mediated pathway (137). The presence of alternative forms of ERα (66-KD): ERα36 (36-KD) and ERα46 (46-KD), which are mainly located in the cytoplasm and plasma membrane, play additional roles and ERα36 has also been associated with tamoxifen resistance (138).

GPER is activated by E2 and it represents a transmembrane receptor that modulates E2 actions (139). The activation of EGFRs by E2 mainly occurs via the membrane- bound GPER1 (76). E2 increases phosphorylated MAPK in SKBR3 cells which express the membrane bound GPER1, while phosphorylation remains unchanged in the MDA-MB-231 which do not express GPER1. However, transfecting the protein into MDA-MB-231 leads to activation by E2 (140). These studies show that ER works in concert with the ErbB family of proteins to foster and assist cancer cell proliferation. With GPR30 being a hindrance in the EGFR signaling pathway and attenuating the inhibition of MAP kinases, combination therapy with tamoxifen and GPER inhibitors could lead to novel therapeutic options (70).

Several retrospective clinical studies have shown the significance of growth factor signaling in both de novo as well as assumed endocrine resistance. While it was previously hypothesized that a loss in the expression of ER could lead to tamoxifen resistance, evidence suggests that only three quarter of tumors express the ER (82). Upregulation of growth factor signaling in MCF7 cells, as well as HER2 in patients treated with tamoxifen, suggests a redundancy in the operation and thus the use of combination therapy may be beneficial (82). Data from studies imply that tumors switch from HER2 to ER and vice versa as the preferred signaling pathway, with therapy toward HER2 leading to an activation of the ER pathway and vice-versa (82, 141). The dependence of the two pathways on each other is highlighted in MBC patients who have been treated with an AI or the ER downregulated with fulvestrant and have progressed with trastuzumab or lapatinib. Thus, combination therapy with inhibition of the ER and HER2 axis has proven to show a benefit in dual ER-HER2 positive (HER2+) patients as demonstrated in a phase III study (TAnDEM: trastuzumab in dual HER2+/ER+ MBC). Here, patients treated with trastuzumab plus anastrozole, an AI, showed a benefit in terms of the PFS when compared with the women on anastrozole alone (142). Recently, a study targeted the extracellular domain of HER2, which is responsible for HER2 homo- and heterodimerization. Consequently, disrupting the 16aa sequence within the extracellular domain of HER2 (HER2-ECD-Δ451-466), led to a reduction in the activity of HER2, and switched off signal transduction mediators, such as ERK1/2 and PI3K/AKT (143).

Signal transduction inhibitors are crucial mediators in overcoming endocrine resistant BC. It was reported in 2000 that inhibition of HER2 and MAPK in HER2 overexpressing tamoxifen resistant MCF7 cells reinstated the interdependence of ER with nuclear receptor corepressor (N-CoR), thereby enhancing the action of tamoxifen and abrogating the antioestrogen resistance (144). The findings above are supported by clinical studies where tumors over expressing *HER2* and *AIB1* had a worse outcome with tamoxifen (145). In a recent study done on AI treated patients, it was evidenced that AIB1 played an important role in regulating selective ER transcriptional activity and promoting tumor recurrence (146).

Lapatinib, a dual inhibitor of EGFR and HER2 was used to study its role in prototypes of HER2+ BC cell lines with assumed endocrine resistance, where it restores endocrine sensitivity (147). In a phase III study, a combined treatment with letrozole, an aromatase inhibitor, and lapatinib or letrozole alone, showed a benefit in the combination arm of the study with a significantly higher PFS in MBC with ER-negative/HER2+ tumors (148).

Clinical studies are being done using EGFR inhibitors in isolation or a combination therapy in order to address endocrine resistance. A phase II study using tamoxifen and the EGFR inhibitor gefitinib or gefitinib alone, showed no significant enhancement in PFS in the endocrine naïve or group treated with an AI (149). In a separate Phase II study on ER+ MBC, the combination treatment of anastrozole and gefitinib showed an increase in the PFS over patients treated with anastrozole alone (150).

Acquired HER2 mutations lead to endocrine resistance in a proportion of patients with ER+ MBC. Tumors driven by a *HER2* amplified mechanism are known to be resistant to endocrine therapy (142, 148, 151). A whole genome landscape study identified hotspot mutations in *HER2* (D769Y, L755S, and S310Y) which were more common in acquired endocrine resistant tumors (9). Mutations within the *HER2* gene lead to E2 independence and resistance to the first line therapy, which includes tamoxifen, fulvestrant and palbociclib, a CDK4/6 inhibitor in BC patients. In these patients an ER-directed drug in combination with neratinib [a pan-HER tyrosine kinase inhibitor (TKI)] was an effective therapy (152). Mutations within the *HER2* gene are more common in the ER+ MBC setting and *HER2* mutations favor dimerization with HER3 (ERBB3) (153). This study also noted that inhibition of HER2 and the ER pathways is required for therapy in ER+/*HER2* mutant BCs (153). Neratinib has been approved by the FDA for treatment in the adjuvant setting of BC patients with early stage HER2 amplified disease, based on the ExteNET trial (154, 155). In the phase III ExteNET study, the neratinib arm of treatment showed a benefit in the disease-free survival (DFS) when compared to the placebo arm in early stage HER2+ BC (156).

### 3b. IGF-IR

Insulin-like growth factor-I receptor (IGF-IR) is a protein expressed as a trans-membrane tyrosine kinase and is activated by the stimulatory hormone/ligands insulin-like growth factor-I and -II (IGF-I, IGF-II), resulting in proliferation as well as anti-apoptosis (157). The IGF-I system is implicated in the pathogenesis of BC (158). There are also several lines of evidence that dysregulation of the IGF-I system and enhanced IGF-IR activation are involved in resistance to endocrine therapy and that IGFs play a paracrine/autocrine role in promoting tumor growth *in situ* during tumor progression, depending on the tissue of origin (159).

Upon binding of ligand to its receptor, the IGF-IR, it leads to phosphorylation of insulin receptor substrate-1 and−2 (IRS1, IRS2), which promotes growth by signaling through the MAPK/ERK, PI3K/AKT, and JAK/STAT pathways. Elevated plasma concentrations of IGF-I have been linked to a higher risk for BC (160). Genetic polymorphisms in genes encoding IGF-I have been reported, and may contribute to an increased risk for BC (161, 162). IGF-IR activation leads to phosphorylation of the MAPK-AKT and subsequent activation/phosphorylation ERα (56).

Interdependence between IGF-IR and ERα in BC prototypes is well-studied, and is implicated as a mechanism of antioestrogen resistance (163, 164). A study in EGFR-positive tamoxifen-resistant variants of MCF7 (TAMR) cells, demonstrated reduced expression of IGF-IR protein levels when compared to their wild-type MCF7 cells. However, the phosphorylated IGF-IR protein levels were equivalent in the two cell lines under basal growth conditions, this was due to an increase in the IGF2 expression, which activated both IGF-IR and EGFR (158). A study by Creighton et al., identified a set of genes that were either upregulated or downregulated by IGF-I which represent hyperactive pathways and hormone independence (165). Basically, the IGF-I signature obtained from MCF7 cells presented with a similar observation in clinical BC patients. The profiled tumors exhibiting the IGF-I signature correlated with poor prognosis and was indicative of a poor outcome. In another study, patients with tamoxifen-resistant tumors with higher *IGF1* and *ER*α expression developed tamoxifen resistance after a longer period of time, and tamoxifen-resistant tumors had lower *IGF1* and *ER*α expression compared to tamoxifen-sensitive tumors (166). A separate study using a kinome wide siRNA screen demonstrated a role for the combined inhibition of IGF-IR and the insulin receptor (InsR), where the dual tyrosine kinase inhibitor OSI-906 (inhibitor of InsR and IGF-IR) in combination with fulvestrant inhibited the growth of hormone independent tumors when compared to either drug alone (167). The IGF-IR-specific inhibitors (like AG1024 and AEW541) or an IGF2 neutralizing antibody inhibited basal IGF-IR, c-SRC, AKT and EGFR phosphorylation, and significantly reduced tamoxifen-resistant basal cell growth. Interestingly, AEW541 also inhibited insulin and IGF2-stimulated effects in tamoxifen-resistant cells (168).

### 3c. FGFR Signaling

Binding of FGF ligands to the FGFR receptor promotes receptor dimerization followed by activation of the kinase domain and subsequent activation of the PI3K/AKT, RAS/RAF/MEK1/2-ERK, phospholipase Cγ (PLCγ), and STATs (169). Amplification of the genes located at the 11q12-14 chromosomal loci, which includes *FGFR1* and *FGF ligands 3, 4*, and *19* occurs in more than 10% of BC patients, depending on the specific type of BC (170, 171). A third of patients with *FRFR1* amplified tumors also exhibit amplification of oncogenes like *CCND1, FGF3, FGF4*, and *FGF19* (172, 173). *FRGR1* expression and signaling via FGFR1 is essential to survival of *FGFR1* amplified BC cell lines (174). Apart from gene amplifications, enhanced expression of its ligand and mutations have been verified in the FGFRs from BC patients, which points to multifactorial mechanisms involved in the FGF/FGFR pathway (175). Dysregulated regulation of FGF/FGFR signaling and its control in oncogenic processes is poorly understood, although evidence suggests that aberrant FGFR1 signaling mediates endocrine resistance through the PI3K and MAPK pathways (176). A small molecule inhibitor dovitinib (TKI258) led to antitumor activity in FRFR1 amplified MDA-MB-134 and FGFR2 amplified SUM52 BC cell lines (177). Interestingly, the FGFR1 amplified cell lines MDA-MB-134 and SUM44 were resistant to treatment with tamoxifen (176). Activating mutations of the *FGFR* gene have been shown to have oncogenic potential and driving resistance to endocrine therapy (178, 179).

Amplification of *FGFR1* correlated with short overall survival rates (OSR) in HR+ BC (180). Consistent with these findings, FGFR amplifications are associated with poor prognosis and endocrine resistance in HR+ BC (180–182). Resistance to endocrine therapy upon aberrant FGF/FGFR pathway amplifications/signaling, offers a strong rationale to study the role of FGFR inhibitors. FGFR TKIs offer one the best approaches in targeting tumors with ER+/HER2 negative/FGFR amplified status and some of these are in initial phase clinical trials. Some of the selective pan-FGFR inhibitors are BGJ398 (infigratinib), JNJ-42756493, Debio1347, TAS-120, AZD4547, ARQ087, and BAY1163877 (183). The role of BGJ398 was evaluated and initial reports suggest a promising role in breast cancer progression and metastasis to the lung (184). AZD4547 has shown promise as an anti-tumor drug in combination with an AI (anastrozole/letrozole) in the RADICAL trial (ClinicalTrials.gov Identifier: NCT01791985) (185).

Dovitinib, a non-selective TKI (TKI258) was assessed in a phase II study (ClinicalTrials.gov Identifier: NCT01528345) in combination with fulvestrant in HR+/HER2-negative MBC patients (186). The study was terminated due to low numbers of enrolled patients, although the combination arm of treatment showed promising clinical activity in the FGF pathway-amplified subgroup. Another non-selective TKI, lucitanib (E-3810) is in a phase II trial (FINESSE: ClinicalTrials.gov Identifier: NCT02053636) as monotherapy in ER+, FGFR1-amplified/FGFR1-non-amplified MBC (187). Whole exome sequencing revealed amplification of *FGFR1, CCND1*, and *FGF3/4/19*, all being associated with resistance to letrozole (17). The same study also noted that combined treatment of ER+ *FGFR1/CCND1* co-amplified CAMA1 BC cells with palbociclib, and/or the FGFR1 inhibitor, lucitanib, lead to a reversal in antioestrogen resistance (17). A recent study demonstrated amplification of the *FGFR* gene which led to aberrant FGFR signaling and thus resistance to therapy with an anti-ER drug and a CDK4/6 inhibitor (188). In line with these findings, a phase Ib trial (ClinicalTrials.gov Identifier: NCT03238196) with erdafitinib in combination with palbociclib and fulvestrant is ongoing in ER+/HER2 negative/FGFR amplified MBC.

With regard to mutational studies, FGFR mutations are infrequent in BC, although mutations in the *FGFR* gene have been identified in BC with unknown functional roles (178, 189). Genome wide association studies have identified *FGFR2* as one of the loci associated with BC (190). According to another GWAS study, the four genomic locations associated with BC were rs1219648, rs2420946, rs11200014, and rs2981579, all of which were located on intron 2 of the *FGFR2* gene (191). Genetic fusions of the *FGFR1-3* gene also represent a small proportion of aberrations and a causative agent in BC (178, 192, 193). These studies suggest a prominent role for *FGFR1* amplifications and that all future studies and trials could focus their therapeutic strategy at targeting and inhibiting FGFR1.

### 3d. SYK

The spleen tyrosine kinase (SYK) protein tyrosine kinases are comprised of SYK and ZAP-70, which contain two SH2 domains and C-terminal kinase domains interrupted by two interdomains A and B (194, 195). *SYK* is primarily expressed by a variety of hematopoietic cells ranging from B cells, mast cells, neutrophils to macrophages and functions in proliferation, differentiation and adhesion (196, 197). Here, activation of the specific receptor (B cell receptor) promotes phosphorylation of immune-receptor tyrosine-based activation motifs (ITAMs) and recruitment, autophosphorylation of SYK (198). SYK subsequently modulates its actions by activating several downstream effectors like RAS-RAF-MEK-ERK (199). SYK functions as a tumor suppressor in BC with a reduction in SYK expression being associated with poor prognosis and metastasis (200, 201). In samples from patients, *SYK* expression is lost as the tumor progresses from ductal carcinoma *in situ* (DCIS) to invasive breast cancer (202, 203). However, in various other solid cancers it has tumor promoting activity depending on the association of the cancer with inflammation (204). With quite a few SYK inhibitors in clinical trials for other cancers, its role in ER+ BC associated with inflammation could be studied (205–208).

## 4. Cell Cycle Regulators

BC sensitivity to endocrine treatment is impacted by the activity of both positive and negative cell cycle regulators (209). Studies have shown that overexpression of positive cell cycle regulators like *c-MYC, cyclins E1* and *D*, contribute to the development of endocrine resistance by activating CDKs (210, 211). Investigators have also demonstrated that aberrant *c-MYC* expression can contribute to endocrine resistance by altering the regulation of p21WAF1/Cip1 (210, 212). Consistent with the activity of CDK, downregulation or loss of function of the two G1 checkpoint CDK inhibitors, p21 and p27 (CDKN1A and CDKN1B), is associated with endocrine resistance (213, 214). A separate study surmised a central role for E2F proteins, in that unliganded ER plays a role in the E2 deprived growth of long term E2 deprived BC cells by regulating a transcriptional machinery (215). The same study also identified CDK4, an activator of E2F transcription as a modulator for E2 independent BC cell proliferation and inhibition of CDK4 led to an inhibition in the growth of fulvestrant-sensitive and -insensitive ER+ cell lines in the absence of E2 (215). These findings supported the development of CDK4 inhibitors as possible therapeutics for BC.

The cyclin D-CDK4/6-INK4-retinoblastoma (Rb) pathway plays a pivotal role in the proliferation of normal breast epithelial and cancer cells. The CDKs are crucial cyclin dependent drivers in cell cycle, cell division and thus cancer. Their importance in the cycle is well-known, with the most common being: G_0_ (quiescence), G_1_-phase (pre-DNA synthesis), G_1_/S-phase, S-phase(DNA synthesis), and M-phase (cell division) cyclins (216). There are typically four different CDK's (CDK1, 2, 4, and 6), dependent on the cyclins D (D1, D2, D3) (217–219). Nascent DNA synthesis occurs during the S phase of the cell cycle at which the cyclin D1-CDK4/6 complex serves as the enzyme that catalyzes the phosphorylation of Rb (retinoblastoma) protein and dictates DNA replication. Coupling of cyclin D to CDK4/6 is regulated by the INK4, Cip, and Kip group of proteins. CYCLIN D1 activates the CDK4/6 essential for mediating RB-induced cell cycle progression at the G1/S checkpoint (220, 221). In the TCGA network studies, *CYCLIN D1* is amplified in 58% of luminal B BCs with 25% showing a gain in CDK4. However, *CYCLIN D1* was amplified in only 29% of luminal A tumors with only 14% showing a CDK4 gain (35). In another study, luminal B-type BCs resistant to endocrine therapy were identified by a unique gene signature indicating a loss of RB protein (222). The E2F4 transcriptional cascade is suppressed upon CD4/6 inhibition in hormone independent ER+ BC cells and ER+ BC patients, thereby supporting the benefit of adjuvant CDK4/6 inhibition in ER+ patients (223).

Thus, Cyclin D1 and CDK4/6 inhibitors represent strategies to overcome endocrine resistant BCs and potent CDK4/6 inhibitors have become extensively available in the last decade. A recent study revealed aberrant FGFR signaling as the mechanism causing resistance to combinatorial therapy by anti-ER's with CDK4/6 inhibitors (188).

Preclinical studies have confirmed the usefulness of CDK4/6 inhibitors specific to a particular molecular subtype of BC (224, 225). Three FDA approved, novel CDK4/6 inhibitors used in treatment of ER+ MBC are palbociclib (PD0332991, Pfizer), ribociclib (LEE011, Novartis and Astex), and abemaciclib (LY835219, Eli Lilly) (226–228). In BC cell lines exhibiting the luminal ER+ BC subtype, proliferation was inhibited by palbociclib and ribociclib (225). The BC cell lines exhibiting non-luminal or the basal subtypes, presented with resistance to palbociclib (225). The clinical trials involving the three prominent CDK4/6 inhibitors: PALOMA for palbociclib, MONALEESA for ribociclib and MONARCH for abemaciclib; were studied for efficacy in MBC patients. CDK4/6 inhibitors are used both as a single agent and as a combination therapy. With endocrine resistance being an obstacle toward effective therapy, the addition of a CDK4/6 inhibitor to endocrine therapy as a combination therapy presents with prolonged PFS and has recently been included as a first line therapy in advanced HR+ BC (226, 228–230) (Figure 3B).

### 4a. Palbociclib (PD0332991)

In the largest clinical validation of a CD4/6 inhibitor, palbociclib was seen as a breakthrough therapeutic drug. In the phase II PALOMA-1 trial on ER+/HER2 negative advanced BC patients, significant prolongation of median PFS was demonstrated in the combination arm (palbociclib with letrozole) when compared to letrozole alone (231, 232). Again, the PALOMA-2 phase III study, validated on ER+/HER2 negative MBC patients reported significant benefit in PFS in the combination arm when compared to letrozole alone (226). In the PALOMA-3 phase III trial, a benefit in the PFS was demonstrated for women with ER + MBC in the combination arm (palbociclib with fulvestrant) when compared to fulvestrant alone (233, 234). The PALOMA-4 trial (ClinicalTrials.gov Identifier: NCT02297438) is ongoing as a first line treatment. A summary of the three PALOMA studies, pointed to palbociclib plus letrozole as the therapeutic first choice in ER + MBC, followed by palbociclib plus fulvestrant as another therapy of choice in ER + MBC, previously on an aromatase inhibitor. The Food and Drug Administration (FDA) granted approval for palbociclib to be used as a first line therapy in combination with an AI or letrozole in post-menopausal women with HR+/HER2 negative advanced or MBC. It has also been approved as a combination therapy with fulvestrant in the treatment of HR+/HER2-negative advanced or MBC in women with disease progression following endocrine therapy.

### 4b. Ribociclib (LEE011)

Ribociclib is another selective and reversible CDK4/6 inhibitor approved by the FDA. In the phase II MONALEESA-1 study on HR+/HER2-negative BC patients, a decrease in the expression of the cell proliferation marker Ki-67 was observed in the combination arm (letrozole with ribociclib) and single agent arm (letrozole alone) (235). The phase III MONALEESA-2 trial was a crucial trial focused as a first line treatment in untreated post-menopausal women with HR+/HER2-negative recurrent or MBC. The results from this trial presented with an extension in the PFS for the cohort of patients treated with ribociclib plus letrozole when compared with the placebo group of letrozole alone (228). In the subsequent phase III MONALEESA-7 trial, appreciable benefits were observed in HR+/HER2-negative advanced BC patients. Here too, the combination arm (ribociclib with tamoxifen/AI and goserelin) showed a 2-fold enhancement in the PFS when compared with the placebo arm (236, 237). Based on the results from the MONALEESA trials, the FDA approved ribociclib in combination with an AI as a first line therapy in pre/perimenopausal women with HR+/HER2-negative advanced or MBC. It was previously approved as a first line therapy or following disease progression on endocrine therapy in combination with fulvestrant or an AI in post-menopausal women with HR+/HER2-negative advanced or MBC.

### 4c. Abemaciclib (LY2835219)

Abemaciclib is another CD4/6 inhibitor, although it has higher selectivity and inhibitory effect for CDK4 than for CDK6 (238–240). In the phase II MONARCH-1 trial, abemaciclib showed enhanced clinical benefit as a single agent in refractory HR+/HER2-negative MBC following multiple prior therapies (241). The phase III MONARCH-2 study conducted on advanced BC patients, demonstrated an extension in the PFS in the combination arm (abemaciclib with fulvestrant) over the placebo arm (fulvestrant alone) (242). Again, the phase III MONARCH-3 study, of abemaciclib (CDK4/6 inhibitor) plus an AI (letrozole or anastrozole) compared to letrozole/anastrozole alone on HR+/HER2-negative MBC has shown promise as an initial therapy with significantly improved PFS (227, 243). Based on the results from the MONARCH trials, the FDA approved abemaciclib as a combination therapy with an AI in post-menopausal women with HR+/HER2-negative advanced or MBC, as a combination therapy with fulvestrant in women with HR+, HER2-negative advanced or MBC with disease progression following endocrine therapy and as a monotherapy for women and men with HR+/HER2-negative advanced or MBC with progression following endocrine therapy and prior chemotherapy.

The impressive and exceptional results from the CDK4/6 inhibitor trials have led researchers to study its efficacy and suitability in several other trials using the CDK4/6 inhibitor as a single agent and in early stage BC (244, 245). The essence of a role for CDK4/6 inhibitors stems from the need for novel agents to supplement endocrine therapy in high risk ER+ disease. Several other trials like the PEARL, NeoPalAna, neoMONARCH, FELINE, CORALLEEN, PELOPS, KENDO (ClinicalTrials.gov Identifier: NCT03227328), and SONIA (ClinicalTrials.gov Identifier: NCT03425838) trials, all are focused toward evaluating the efficacy of CDK4/6 in the advanced ER+ BC and in other BC subtypes (245).

### 4d. Combination of CDK4/6 With PI3K/AKT/mTOR

The remarkable results observed with CDK4/6 inhibitors has been very well-received and approved by the FDA. However, a large number of ER+ BC patients continue to experience relapse and recurrence (246). There is a complex crosstalk between ER+ BC, constitutive PI3K activation and the CDK4/6 pathway. This provides a strong rationale for the combined targeting of both the CDK4/6 and PI3K pathways in effective control of the tumor progression (247–249). Studies have suggested that inhibition of the either the CDK4/6 or the PI3K/AKT pathway, delays the onset of endocrine resistance (248, 250). Combinatorial inhibition of both the PI3K and CDK4/6 pathways overcomes the resistance due to single agent inhibition by CDK4/6 by downregulating cyclin D1 and arresting cell cycle progression (248, 249). Several combination therapy trials are underway to identify the best strategy to overcome endocrine resistance in ER+ BC. Trials that combine the CDK4/6 inhibitors with various PI3K/AKT/mTOR inhibitors (ClinicalTrials.gov Identifiers: NCT03128619, NCT03006172, NCT02684032, NCT02732119, NCT02871791, NCT02599714) are ongoing, with the aim to inhibit tumor growth and prevent relapse. The triplet inhibitor trial (ClinicalTrials.gov Identifier: NCT02088684) with ribociclib, fulvestrant and BKM 120 (buparlisib, PI3K-pan class I-inhibitor) or BYL719 (alpelisib, PI3K-alpha specific class I inhibitor) in HR+/HER2-negative advanced or MBC is one study aimed to study its efficacy and suitability as a mode of treatment in these patients.

### 4e. Combination of CDK4/6 With Immune Checkpoint Inhibitors

CDK4/6 inhibitors act as cell cycle checkpoint inhibitors and induce cell cycle arrest, senescence and immunogenicity in the TME. Studies have demonstrated an enhancement in the anti-tumor response upon treatment with CDK4/6 inhibitors due to an enhancement in interferon production, reduced T-regs and enhanced T cell activation (251, 252). Addition of immune checkpoint inhibitors (PD-1and CTLA-4) to CDK4/6 inhibitors holds promise by inhibiting the growth and proliferation of cancerous cells and, with this study as the background, a clinical trial (JPCE) has focused on studying the suitability and efficacy of using abemaciclib in combination with pembrolizumab in HR+/HER-2 negative metastatic BC patients (253). Another study is recruiting to study the response of fulvestrant and pembrolizumab as a combination therapy (ClinicalTrials.gov Identifier: NCT03393845) in HR+/HER-2 negative advanced or metastatic BC patients.

### 4f. CDK4/6 Associated Response and Fusion Genes

Although CDK4/6 inhibitor are effective in controlling tumor growth, a fraction of the cells acquire resistance to CDK4/6 inhibitors. Upon mechanistic dissection, a role for the FAT1 and RB1 proteins were identified, wherein, a loss of function in FAT1 was observed with a concomitant increase in CDK6 which was attributed to enhanced binding of YAP and TAZ on the CDK6 promoter and regulation of the Hippo pathway and associated factors within the pathway (254).

High throughput RNA sequencing (RNA-seq) methods have led to the identification of *ER*α gene fusions with some instances of more than four *ER*α coding exons fused in frame or out of frame (17, 104, 255). A number of *ER*α gene fusions have been identified in endocrine resistant and ER+ MBC, some of the common ones being the *ER*α*-e6* > *DAB2, ER*α*-e6* > *GYG1* and *ER*α*-e6* > *YAP1* structures (104, 256). Li et al. in 2013, identified an in-frame *ER*α fusion protein consisting of *ER*α exons 1–6 and the c-terminal YAP1 sequence (*ER*α*-e6* > *YAP1*) that functioned as a driver of endocrine resistance and E2 independent proliferation (104). A recent study by the same group identified at least two fusion genes (*ER*α*-e6* > *YAP1* and *ER*α–*e6* > *PCDH11X*) in advanced ER+ BC and palbociclib was observed to inhibit the fusion gene driven T47D growth under hormone deprived conditions in a dose-dependent manner (257). This observation was consistent with previous reports of palbociclib being able to suppress the growth of pRb expressing tumors with *ER*α mutations under E2-deprived conditions (258).

A genetic landscape sequencing study on circulating tumor DNA of samples from the PALOMA-3 study demonstrated that acquired mutations in the *RB1, PIK3CA*, and *ER*α genes emerged in the treatment arm of fulvestrant and palbociclib (259). In this study too, *ER*α Y537S was one of the driver mutations that promoted resistance to fulvestrant and clonal evolution is surmised as the principle behind resistance to therapy (259).

## 5. Other Transcription Factors

### 5a. PI3K/AKT/mTOR

The PI3K/AKT pathway is mainly activated downstream of RTKs, which can phosphorylate several targets, including NFκB, BAD, IKK, p27, FOXO1, GSK-3β, MDM2, and mTOR, with the mTOR complex being the best described target of AKT signaling (260). The tumor suppressor gene phosphatase and tensin homolog (PTEN) is a negative regulator of mTOR pathway (261). PTEN may be downregulated through several mechanisms, including mutations, loss of heterozygosity, methylation, aberrant expression of regulatory microRNA, protein instability and activated mTOR signaling is also associated with Cowden's syndrome (*PTEN* mutations) (262).

PI3K is comprised of several isoforms of the regulatory (p85) and a catalytic subunit (p110). Mutations are frequently identified in BC within the *PIK3CA* gene, encoding the p110α catalytic subunit (263). A large number of the *PIK3CA* mutations occur within the kinase (H1047R) and helical domains (E542K and E545K) of p110α subunit (264, 265).

The PI3K/AKT and E2 signaling crosstalk has been studied extensively in BC. As previously mentioned, activated AKT can phosphorylate ERα at serine-167 in the AF-1 domain and increase ERα-dependent transcription (56). Activation of the PI3K pathway in breast tumors is associated with reduced ERα levels and endocrine resistance (266). Signaling via GPER is another mode of signaling utilized by E2 activated PI3K (p110α subunit) which leads to inactivation of FOXO3a, thereby promoting progression of BC (74). GPER antagonists in combination with a SERM/SERD therapy could serve as an effective therapeutic strategy in GPER positive/ER+ BC (74). The above studies have also shown the importance of a crosstalk between the PI3K and ER pathway in antioestrogen resistance and suggested that combining a PI3K inhibitor with an ER down regulator is more effectual than either of them alone (267). Thus, constitutive activation of GFR signaling pathways leads to reduced ERα levels and subsequent endocrine resistance. In contrast, inhibition of the pathways leads to sensitization in response to antioestrogens and other oncogenic pathways are activated upon E2 deprivation or inhibition of ER in the endocrine resistant scenario (267).

Two other interdependent factors, mTORC1 and mTORC2 are part of a positive feedback mechanism belonging to the PI3K pathway, which have independent regulatory mechanisms and exert their effects through distinct targets and mechanisms (268). Upon inhibition of mTORC1, the alternate complex mTORC2 leads to an activation of the PI3K pathway, which indicated that inhibition of one arm of the pathway (via inhibitor), led to activation of another arm and may not be sufficient to produce a clinical benefit (269).

Clinical studies have validated the association between activation of the PI3K and *de novo/*assumed resistance to endocrine therapy. Several trials have been done where inhibitors of PI3K pathway have been combined with endocrine therapy. In a neoadjuvant study by Baselga et al., treatment with letrozole and everolimus (mTOR inhibitor), a more pronounced reduction in tumor cell proliferation and improved clinical response was observed when compared with letrozole alone in patients with early-stage ER+ BC (270). In another Phase III combination study, also called the BOLERO-2, patients with ER+ advanced or MBC, were either given everolimus and exemestane (an AI), or exemestane and placebo. The combination of everolimus and exemestane was found to have a median PFS that was significantly much superior to the exemestane only arm (271). Due to highly successful rates of PFS, this combination therapy of exemestane and everolimus has been approved on selected advanced patients of ER+ BC in the USA and Europe. A recent study has produced a systematic approach and an effective rationale behind the use of an mTORC1 inhibitor (RAD001) in combination with neratinib and tamoxifen/fulvestrant to target BC patients who relapse due to endocrine resistance, for example patients treated with RAD001 and an AI (272). This study demonstrated that triple blockade with RAD001, neratinib and tamoxifen/fulvestrant was highly effective in the long term E2 deprived BC cell lines and was well-tolerated in a xenograft model (272).

The BOLERO-2 study also demonstrated that patients who has the D538G and Y537S mutation within the ERα gene presented with aggressive disease and a shorter OS. When considering the benefit based on mutation site, it was more pronounced in the D538G group due to a larger number of patients. In the TAMRAD phase II trial, patients with ER+ MBC becoming resistant to AI treatment were given tamoxifen and everolimus or tamoxifen alone. Here too, patients in the combination arm showed an improved clinical benefit rate, time to progression, as well as an overall survival compared to patients treated with tamoxifen alone. This study stratified the patients based on primary and secondary endocrine therapy resistance and interestingly, patients with relapse after 6 months of AI treatment presented with an improvement in the PFS when compared to those patients that relapsed during adjuvant AI before 6 months on the treatment (273). Thus, further studies are warranted where a proportion of patients could benefit from the combined mTOR/ER targeting approach based on specific mutations within the ERα gene.

In the phase 3 BELLE-2 trial, patients with ER+/HER2 negative, locally advanced or MBC who had progressed on or after AI treatment and had received at least one line of chemotherapy treatment, were given the pan-PI3K inhibitor buparlisib plus fulvestrant or fulvestrant alone and it was observed that the combination arm of the treatment showed improvement in the PFS in these patients, although the toxicity observed warrants the use of other more α specific PI3K inhibitors (274). With the focus on *PIK3CA, PIK3CB*, and *PIK3CD* mutant tumors, some of the more recent isozyme-specific inhibitors target the p110α, p110β, and p110δ isoforms of PI3K: BYL719 (alpelisib) and MLN1117 for p110α and GDC-0032 (taselisib) for 110β-sparing inhibitors (275). In the phase III SOLAR-1 study (ClinicalTrials.gov Identifier: NCT02437318), post-menopausal women with HR+/HER-2 negative advanced BC were given either alpelisib/placebo (a PI3Kα specific inhibitor) in combination with fulvestrant and a significant extension in the PFS was reported in patients with *PIK3CA* mutations in the alpelisib arm of the study (276).

### 5b. PARP

Poly (ADP-ribose) polymerase (PARP) is a family of nuclear proteins which plays a vital role in the recognition and repair of endogenously/exogenously induced DNA damage (277, 278). PARP like BRCA1 and BRCA2 acts by repairing DNA and PARP inhibitors act by disrupting the DNA repair mechanism and increasing genomic instability (279, 280). Basically, it is the synthetic cellular/DNA lethality caused due to *BRCA1/2* mutation along with PARP inhibition that leads to a block in the DNA repair pathway and subsequent death/apoptosis of the *BRCA* mutated/deficient cells (281, 282). The two PARP inhibitors approved by the FDA are olaparib and talazoparib, with talazoparib having a higher potency than olaparib due to a mechanism of action called DNA trapping (283, 284). The PARP inhibitor olaparib has shown promise as a drug of choice in germline BRCA-mutated, ER+/HER-2 negative MBC patients who have been treated with a prior endocrine therapy or been considered inappropriate for endocrine treatment (285). The decision was based on the results from the OlympiAD (ClinicalTrials.gov Identifier: NCT02000622) study conducted on MBC patients with a germline *BRCA1/2* mutation where there was an OS benefit among patients who had not received chemotherapy for MBC (285). Based on the EMBRACA study (ClinicalTrials.gov Identifier: NCT01945775) on *BRCA* mutated subjects with locally advanced and/or MBC, talazoparib provided benefit in the PFS over physician's choice of chemotherapy (286). The use of PARP inhibitors holds promise in *BRCA1/2* mutated ER+ MBC patients in combination with other appropriate therapies. Another viewpoint is that activation of CDK4/6 when in complex with other cyclins leads to the phosphorylation of RB1 which blocks binding the binding of RB1 and repressing E2F regulated genes (287). PARP1 protein is reduced by the recruitment of E2F1-RB1-HDAC1-EZH2-SWI/SNF complex in hematopoietic stem cells and monocytes (288). Reduction/loss of PARP1 leads to an impairment in the 8-oxoguanine glycosylase (OGG1)-dependent DNA repair mechanism and an arrest at the G1 phase in PARP1 overexpressing cells, leads to the formation of a dense complex with OGG1 (PARP1-OGG1 complex) (289). In oxidatively stressed cells, CDK4/6 inhibitors also act by repressing PARP and causing an aberrant DNA repair mechanism (289). There is a functional complex between CDK4/6, RB1, PARP1, and OGG1 (289). However, with tumors eventually acquiring resistance to PARP inhibitors, the therapeutic advantage/possibility of combining CDK4/6 inhibitors and PARP inhibitors offers hope(290).

### 5c. MAPK/ERK

Activation of the RAS/RAF/MEK1/2-ERK pathway leads to phosphorylation of ERα at serine 118, resulting in cell proliferation (291, 292). The RAS/MAPK/ERK pathway increases sensitivity of ERα to low concentration of E2 leading to endocrine resistance (144, 293, 294). Tamoxifen resistant (TAMR) BC cells show increased levels of activated MAPK and ERα (295). McGlynn et al., have shown an association between increased expression of activated Raf-1, pRaf (ser338), MAPK and a greater risk of relapse following treatment with tamoxifen in clinical samples (296). ERα is activated following phosphorylation by MAPK at serine 118 in the AF1 domain (297) and previous studies have demonstrated that inhibiting the MAPK/ERK pathway leads to an upregulation of ERα and subsequent re-sensitization to tamoxifen (298). Another study has shown that tamoxifen resistance is mediated through CDK10 suppression by activation of the MAPK/ERK1/2 pathway, thereby overcoming the dependence upon ERα signaling (299). The active RAS-GTP to converted to inactive RAS-GDP through NF1, a large transcriptional protein, which acts as a tumor suppressor (300).

A negative regulator of the RAS pathway, NF1 is associated with an increased risk of BC (301, 302). Independent Component Analysis (ICA) elucidated the association of NF1 and NFAT to clinical outcomes in BC (303). A lack of NF1 type 1 expression in the aggressive endocrine resistant MDA-MB-231 BC cell line was associated with an increase in phosphorylated MAPK and activated RAS (304). NF1 mutations have been identified in luminal or ER+/HER2-negative, HER2-enriched and triple negative BC (35, 300, 305). NF1 was one among the compendium of genes whose silencing led to tamoxifen resistance in MCF7 cells (306). A recent report revealed mutually exclusive hotspot mutations in NF1, ERBB2 and *ER*α that were acquired after endocrine therapy and play a role in endocrine resistance (9). Since the RAS/RAF pathway is induced by both ERBB2 (gain-of function) and NF1 (loss-of function), an in-depth analysis of the pathway alternations before and after endocrine therapy, revealed hotspot mutations in *KRAS, HRAS, BRAF*, and *MAP2K1* (*MEK1*). Oncogenic mutations were present in more than one of the RAS/RAF pathway effectors that did not present with an *ER*α mutation in the post-hormonal HR+/HER2-negative tumors (254). Additionally, targeting the RAS/RAF pathway with an ERK inhibitor SCH772984 re-sensitized MCF7-EGFR cells to fulvestrant (9). Targeted sequencing was performed to study the prognostic effects of somatic mutations and it was reported that *NF1* frame-shift nonsense *(FS/NS)* mutations has adverse effects on prognosis (19). A recent study identified an enrichment in NF1 alterations in metastatic Invasive Lobular Carcinoma (mILC) and a role for NF1 in endocrine resistance (307). The RAS/RAF pathway along with NF1 are therefore involved in crosstalk between ERα and RTK's, and are associated with tamoxifen resistance (297).

### 5d. c-SRC/Kinase

c-SRC, a non-receptor tyrosine kinase is associated with the progression of many human cancers, including BCs (308). Src-1 (NCOA1) serves as a master regulator, a transcriptional hub that complexes with AP1, NF-κB, p300/CBP and other co-activators to activate the ER and PR (309, 310). Basically, SRC-1 interacts with AIB1, ETS2 and HOX11 to define its association in BC progression and endocrine resistance (311, 312).

Studies have shown an activation and upregulation of SRC associated with acquiring tamoxifen resistance in ER+ cell lines (313). SRC is involved in various signal transduction pathways including ER and HER2/EGFR (314). Elevated levels of c-SRC may be due to overexpression of growth factors (315, 316). c-SRC is involved in various oncogenic signaling pathways including growth, invasion and metastases (317, 318). The interaction between ER and SRC is well-established and they form complexes with an array of proteins including PI3K, FAK, PELP, and MNAR leading to the activation of pAKT and pMAPK demonstrating the role that c-SRC has in endocrine resistance (318–323). A recent study demonstrated the effect of TGF-β, where it promoted epithelial mesenchymal transition (EMT) and expression of the EGFR and IGF-IR. These RTKs (EGFR and IGF-IR) formed complexes with ERα and SRC, thereby supporting endocrine resistance in BC (324). Furthermore, c-SRC phosphorylates the ER and has been shown to regulate ER localization (325, 326). Upon treatment with tamoxifen, c-SRC activity is increased and this subsequently amplifies the extent of invasion and motility in BC cells (313). Moreover, since c-SRC is essential in modulating tamoxifen resistance, and blocking its activity reverses tamoxifen resistance (327). c-SRC inhibitor could be exploited as a combinatorial therapeutic drug in the treatment of human BC.

### 5e. STATs

The STAT-family members (STAT1-4, STAT5a, STAT5b, and STAT6) represent a family of transcription factors involved in cell proliferation, differentiation, survival and apoptosis. They have all been shown to be expressed in BC cell lines, but only STATs 1, 3, 5a, and 5b are expressed in BC tissues (328, 329). It is well-understood that the STAT proteins mediate cell proliferation and survival by regulating and influencing the activities of several other transcription factors and associated pathways. It has been shown that the STAT3 and STAT5 signaling pathways are integrally involved in endocrine resistance and more so in the growth factor-stimulated cases. Also, there is functional redundancy between the two STAT proteins, where one protein overcomes the loss/gain in function due to the others gain/loss in function (330, 331). The activation of STAT3 and STAT5 pathways are downstream of the EGFR and c-SRC pathways, which present as being overexpressed in tamoxifen resistant tumors. SRC-1 also interacts with STAT1, STAT3, STAT5 and STAT6, and although STAT3 and STAT5 are considered as tumorigenic transcription factors, STAT1 is a tumor suppressor (309, 332–334). It has also been demonstrated that ER+ BC cells treated with tamoxifen, results in decreased phosphorylation of STAT3 at serine 727, suggesting an association between tamoxifen sensitivity and decreased STAT3 transcriptional activity (335). Yamashita et al. in 2006 had shown that STAT5 is a strong prognostic molecular marker in ER+ BC. In their study, they investigated the expression of STAT3 and STAT5 in more than 500 BC tissues by immunohistochemical techniques and observed that in ER+ patients with STAT5 positive tumors there was significantly increased overall survival, thereby suggesting that expression of STAT5 is helpful in selecting patients who could possibly benefit from endocrine therapy (336). Recently, a role for increased STAT1 signaling in endocrine resistance was reported in studies that identified STAT1 as a potential mediator of endocrine resistance/sensitivity in BC and an appropriate STAT1 inhibitor could serve as a therapy in endocrine resistant BC (331, 337).

### 5f. NF-kB

The NFκB family of transcription factors is comprised of five members (RelA/p65, RelB, cRel, NFκB1/p50, and NFκB2/p52), all of which play an important role in cellular homeostasis (338). Accumulating evidence in support of NFκB activation in cancerous stem cells has led researchers to focus on various genetic signatures (339). It not only creates a microenvironment suitable for stem cell survival, but also its invasiveness and metastatic capability. A line of evidence supports the notion that NFκB and CXCR4 helps to maintain the stemness and promotes migration of cancerous cells (340). Increased expression of p50/NFκB1, p52/NFκB2, and c-Rel was detected in breast tumors compared to adjacent normal tissue (341). Activated NFκB activity has been detected in hormone resistant BC cells and RelA/p65 expression was upregulated (342). A separate study demonstrated that upon inhibition of NFκB and thus also the RelA/p65 by parthenolide (an NFκB inhibitor), sensitivity to tamoxifen was restored in resistant MCF7 cell lines, along with decreasing BCL2 expression, which can be reversed by a caspase 8 (CASP8) specific inhibitor (343). Inflammatory molecules leading to endocrine resistance occurs through kinases that regulate ERα directly or through ERα and RelA/p65/NFκB complexes at ERE enhancer sites to either upregulate or downregulate respective genes (344).

A previous study showed that the expression and DNA binding of NFκB1/p50 and NFκB2/p52 were enhanced in LCC1 estrogen-independent, TAM-sensitive BC cells compared to MCF-7 estrogen-dependent cells, which further demonstrated a role for NFκB in the pathway to endocrine resistance (345). Gionet et al. reported that NFκB1/p50 binds to the ERα on EREs and inhibiting NFκB increased the expression of E2 responsive genes (346).

A separate study suggested that NFκB induces breast cancer progression by stimulating IL-6 and IL-8 (347, 348). Another study reported that FOXA1 led to suppressed *IL6* expression by disrupting the binding of NFκB to the IL-6 promoter and thus concluded that reduced *FOXA1* expression leads to cancer stem cell like properties in tamoxifen resistant cells through the preferential binding of NFκB to the IL-6 promoter and upregulating IL-6 expression (349).

### 5g. LMTK3

Lemur tyrosine kinase 3 (LMTK3) is a serine-threonine-tyrosine protein kinase involved in various cancers. Aberrant expression of the gene and polymorphisms within the gene serve as suitable biomarkers in cancer progression (350, 351). LMTK3 is expressed in both ER+ and ER-negative BCs and a kinome screen identified it as an ERα regulator (352). In an elaborate experimental setup, a role for LMTK3 in endocrine resistance was confirmed (353). Future studies could focus on its use as a valuable biomarker and therapeutic target.

## 6. Hypoxia Inducible Factor

The intra-tumoral pressure of oxygen (O_2_) serves as an important indicator of the possibility of tumor metastasis (354). In BC, hypoxia is induced due to reduced oxygen levels becoming available at the site of the tumor due to their distance from functionally viable blood vessels, and it in this simmering tumor conducive and expansive microenvironment that activation of its main effector, hypoxia-inducible factors (HIF) takes place. The HIF family of transcription factors are comprised of at least three factors: HIF1, HIF2, and HIF3, each of which exists as a heterodimer, with an O_2_ inducible α and a constitutively expressed β subunit (355). Hypoxia associated HIF1α is quite commonly coupled with errors in DNA replication including homologous replication and high mutation rates along with changes in gene expression which is mainly due to epigenetic changes regulated by histone demethylases.

Mounting evidence suggests that a large proportion of the HIF1α targets are also targets of ERα and that the *HIF1*α gene is endowed with the presence of an ERE within its genomic architecture and is regulated by ERα (356). The transcription factor HIF1α is regulated by ERα and that increased *HIF1*α expression confers endocrine resistance to ERα+ cancer cells. Clinical studies have thus shown that HIF1α is associated with endocrine resistant BC (356). One of the common target genes for both the ERα and HIF1α is *KDM4B/JMJD2B*, which is a H3K9me3/me2, H3K36me2/me3, H1.4K26m2/me3 histone demethylase and its genomic landscape contains binding sites for both HIF1α and ERα (356). It has also been demonstrated that both KDM4A and KDM4B form a complex with ERα and lead to the regulation of ERα target genes and an upregulation of both the genes has been observed in both ERα+ and ERα- cells (357). Another histone demethylase of the KDM4 family: KDM4C, promotes BC cells proliferation and metastasis by serving as a transcriptional activator of HIF1α (358). HIF1α promotes angiogenesis within tumors via regulation of its target gene VEGF, thereby leading to BC cell proliferation and metastasis. Hypoxia is the principal regulator of VEGF expression, as it is a direct transcriptional target of both HIF1α and HIF2α (359).

Accumulating evidence from preclinical and clinical studies have demonstrated a role for HIF1α in endocrine resistance. Endocrine resistance was observed in MCF7 cells transfected and stably expressing HIF1α, and targeting it with zoledronic acid led to endocrine sensitivity (360). Signaling via the GPER-HER2-ERK-cFOS pathway triggers HIF1α dependent VEGF activation and BC progression (73). An enhancement in the resistance of BC cells to tamoxifen and fulvestrant was observed in ERα+ cancer cells transfected with HIF1α (356). A recent study reported that HIF1α and p44/42MAPK play an important role in endocrine resistance and may serve as appropriate therapeutic targets for endocrine resistant patients treated with an AI (361, 362).

## 7. Stem Cell Population and Transcription Factors

Cancerous/tumorous stem cells give rise to tumors/cancers and define their ability to proliferate and metastasize. They encompass the ability to regenerate or self-renew and initiate cancer progression, proliferation, migration and metastasis. The stemness of these cells are correlated with poor prognosis and endocrine resistance. A few of the biomarkers that define these subpopulations are CD44, CD24, CD133, and ALDH1 along with others (363).

A recent study identified FOXA1 as a prominent factor for ERα activity in breast cancer (364–366). Induction of FOXA1 triggers a cascade of events leading to endocrine resistance, one of the most prominent being the induction of IL-8 by the FOXA1 expressing ER+ tumors (364). Another study identified the FOXM1 motif bound strongly to the ERα DNA in endocrine resistant cells by expansion of BC stem cells and could be a promising target aimed toward BC therapy (367). RUNX1 has been identified as a BC stemness repressor by repressing *ZEB1* expression (368). RUNX2 is another stemness gene and a separate study identified that RUNX2 and ERα interact with each other in an ER+ endocrine resistant BC cell line to mediate EMT and metastasis, and supports a role for RUNX1 in endocrine resistance (367). Interestingly the same study also highlighted the upregulation of SOX9 in tamoxifen resistant clinical samples. RUNX1 is a tumor suppressor gene that has recently been identified to have point mutations in ER+ BC (369). Xue et al. identified FXYD3 as a target of the E2-ER pathway and it is upregulated in the ER+ BC stem cells (370). They also went on to validate its importance in ER+ BC cells. Crucially, the study identified a direct regulation of the FXYD3 by SOX9.

There are possibly many transcription factors and associated pathways that may transform a normal stem cell to a cancerous stem cell, although two of the well-defined ones are: NOTCH and Hedgehog (Hh). The NF-kB pathway has been described elsewhere.

### 7a. NOTCH

The 4 NOTCH transmembrane receptors: NOTCH 1–4, interact with multiple ligands (Delta-like 1, Delta-like 3, Delta-like 4, Jagged 1, and Jagged 2), resulting in cleavage of the intracellular domain by γ-secretase. The intracellular domain then translocates to the nucleus where it binds with co-activators to regulate transcription of target genes and thereby plays an important role in endocrine resistance (371). NOTCH signaling has been shown to play a critical role in normal human mammary development and also regulation of cancer stem cells in both invasive carcinoma of the breast as well as ductal carcinoma *in situ* (372, 373). Augmenting the activity of NOTCH4 in BC cells led to an increase in the expression of BC stem cell biomarkers and the higher expression of NOTCH in these cells, were evident in both the basal and luminal cell types (374). Another study went on to delineate the role of NOTCH1 in inhibiting tumor progression in BC, whereby inhibition of NOTCH1 resulted in tumor regression and prevention of recurrence in ~67% of the tumors studied (375). It has recently been demonstrated that NOTCH pathway is hyperactivated in resistant BC cells and can be abrogated by blocking the NOTCH pathway (376). Previously, it was shown that E2 inhibits NOTCH activity by affecting the NOTCH receptor cellular localization and that tamoxifen and raloxifene blocks this effect, thereby activating the NOTCH pathway. Inhibiting the NOTCH signaling pathway with γ-secretase inhibitors was more effective when used with tamoxifen (377). Recently, it has been reported that Nicastrin, an essential subunit of gamma secretase, and NOTCH4 are key molecules involved in resistance to endocrine therapy and that the gamma secretase inhibitor (GSI) PF03084014 and anti-Nicastrin MAbs can possibly reverse and potentially re-sensitize endocrine resistant BCs (378).

In BC, SOX2 was identified as a factor responsible for the reprogramming of BC cells into BC stem cells and its enhanced expression was associated with the HER2+ BC subtype (379). SOX2 is activated by the NOTCH signaling pathway and Oct4 localizes β-catenin within the nucleus and thus a role for the NOTCH (described later) and WNT pathways in defining the stemness of BC cells (379, 380). The two pathways interact among themselves, regulating each other's functions and *HES1*, a NOTCH target gene, is regulated by the WNT signaling pathway (381). WNT signaling has been reported to play an important role in BC stem cells (382).

With STAT3 and STAT5 being constitutively activated in cancers, they have also been shown to defy each other's physiological actions with differential effects (383, 384). That NOTCH activated STAT3 pathway plays an important role in endocrine resistance, was verified in an endocrine resistant model of MCF7 cells (TAMR-MCF7 cells), whereby constitutive activation of STAT3 was observed and inhibition of the NOTCH signaling pathway by a NOTCH inhibitor, DAPT led to a concomitant reduction in the level of activated STAT3 observed in this model of endocrine resistant MCF7 cells (385).

### 7b. Hedgehog (Hh) Signaling

The Hedgehog (Hh) signaling pathway has been studied extensively in the past as an essential pathway dictating the initiation, progression and metastasis of cancers, although more recently the focus on this pathway is toward it being at essential crossroads as part of the BC stem cell circuit of pathways (386). This pathway is mainly activated in tamoxifen-resistant tumors and it is activated by the PI3K/AKT pathway (387). A recent study suggested the combined therapy targeting both the PI3K/AKT pathway along with the Hh pathway to tackle endocrine resistance in BC (388). Although the stem cell biomarker CD133 has not been clearly defined, it is a moiety that binds cholesterol and has a prominent role in Hedgehog (Hh) signaling (363). The Hedgehog signaling pathway is regulated by the E2 related receptor β and affect the downstream targets (389).

## 8. Other Factors and Proteins

There are several other transcription factors and proteins that determine cancer progression in the face of ongoing endocrine therapies, some known and many unknown. The EMT factors, Twist and Snail, have been implicated in endocrine resistance, with Twist downregulating ERα by epigenetically regulating the promoter of ERα (390–393). There are several other proteins and molecules that play a major role in endocrine resistance, including the NOTCH and WNT pathways described earlier. All these proteins and transcription factors function as individual molecules working together for/against the homeostasis of the human cells, to determine the fate of cancer cells.

Metabolic and oxidative stress are a few of the other factors affecting endocrine resistance. The mechanisms are elucidated in detail below for each of these factors. Recent publications have demonstrated that homeostasis in cell metabolism and cell proliferation is mediated by a few other transcription factors and pathways as well. Another mechanism for endocrine resistance that has been suggested relates to the ability of the metabolic enzymes to deliver the active compound. Some studies have reported lower concentrations of BC drugs at the site of the tumor when compared to levels in the plasma and an association with poor outcomes (394). Alternatively, a recent study demonstrated that an elevated concentration of tamoxifen metabolites at the site of the cancer, led to agonist effects (395).

### 8a. Oxidative Stress

Oxidative stress is caused due to an imperfect polarization of reactive oxygen species (ROS) vs. antioxidants. Although this holds true under all disease states that progress toward chronic disorders, it stands out as a cause of major concern in cancers, where there is excess cellular mass burden. Thus, it becomes imperative to also understand the role of toxicity to varied drug dosage/regimens and the associated oxidative stress, in other words: the oxidative stress created due to a sudden imbalance in the microenvironment of the cancer. The polyunsaturated fatty acids present in every cell are metabolized to malondialdehyde (MDA) by the ROS whereas nucleic acids (DNA) targeted by ROS are metabolized to 8-oxo-7, 8-dihydro-2′-deoxyguanosine (8-oxodG) (396, 397). A recent study, demonstrated the possibility of using concentrations of urinary 8-oxodG as a marker to define early stage breast cancer patients (398). Again, a study of serum levels of 8-oxodG, indicated an association with ductal carcinoma (399). Glutathione is another antioxidant that exists as reduced glutathione (GSH) and oxidized glutathione (GSSG), the ratio of which (GSH: GSSG) has been inferred as a marker of oxidative stress (400, 401).

A study revealed that E2 led to an increase in ROS production and an upregulation in the expression of genes involved in oxidative stress, e.g., hemeoxygenase 1 (*HMOX1*). An association with c-SRC phosphorylation was observed in this experimental setting and it was attributed to the oxidative stress induced by E2 (402).

In luminal B breast cancer, the loss of sirtuin proteins, particularly SIRT3, promotes tumorous phenotypes dictated by atypically regulated protein acetylation and thus cells are exposed to oxidative stress. One of the analysis from the loss of SIRT3 showed its association with the development of endocrine resistance in the luminal B BC (403). Tamoxifen builds up in tumors as part of the daily dosage regimen, and leads to an increase in tamoxifen induced oxidative stress. This effect leads to an activation of the protein, Nuclear factor-erythroid 2-related factor-2 (Nrf2) which subsequently activates the anti-oxidant response element (ARE). Thus, Nrf2 and ARE levels could serve as oxidative stress biomarkers in tamoxifen treated BC patients (404). These markers could also be studied for their role in endocrine resistant BC and also provide a rationale to study the role of oxidative stress.

### 8b. Drug Metabolism

The therapeutic drugs used in ER+ BC are administered at a certain dosage between fixed intervals to achieve optimal dosage and efficacy which is detected as plasma half-life. The plasma half-life is dictated by the metabolic rate and subsequent elimination of the drug from the plasma. Among the popular drugs studied in ER+ BC, tamoxifen is among one of the most widely used choice of available treatments and is metabolized to its active forms 4-hydroxytamoxifen (4-OH tamoxifen) and N-hydoxy desmethyl tamoxifen (N-OH desmethyl tamoxifen), which is further metabolized to endoxifen in the liver through the polymorphic cytochrome P450 enzymes (CYP2B6, CYP2C9, CYP2C19, CYP2D6, CYP3A4, CYP3A5), the most effective metabolic enzyme being CYP2D6 (405, 406). Interestingly, the potency of its active metabolite endoxifen is far more effective than tamoxifen in its native form. The active metabolites are degraded for excretion by the UDP-glucuronosyltransferases (UGTs) and sulfotransferase (SULTs) enzymes (407). The genotypic information for the CYPs, UGTs, and SULTs has been surmised as one of the molecular mechanisms that define endocrine resistance.

Differences in the expression and associated mutations in each enzyme (CYPs, UGTs, or SULT's) of the tamoxifen metabolic pathway, would help define the effectiveness of the drug in the individual, leading to the well-known concept of personalized medicine. Studies have demonstrated a role of the genotype (expression profiles and/or polymorphisms in the tamoxifen metabolic genes) along with co-intake of other drugs that could lead to altered effectiveness and/or plasma concentrations and associated half-life. One of the crucial CYP genes, CYP2D6, has several polymorphisms within its gene, and each have been associated with either an increase or decrease in enzyme activity. Each variant defines the metabolic rate of tamoxifen, denoted as either extensive (EM), intermediate (IM), or poor metabolizers (PM) (408, 409). Studies have reported altered plasma concentrations which correlate to polymorphisms in CYP2D6 (406). One of the first studies that investigated the polymorphisms in CYP2D6 and SULT1A1 in 226 BC patients on tamoxifen, presented with differences in recurrence of the disease based on the homozygosity/heterozygosity of the CYP2D6^*^4 and SULT1A1^*^1 alleles (410).

In another study conducted by a Korean group (Asian cohort), a polymorphism at CYP2D6^*^10/^*^10 correlated with lower plasma concentrations of the tamoxifen metabolites (411). The variation between activities of the CYP2D6 gene due to allelic variants across various ethnicities becomes imperative when translating preclinical data into clinical use for dosage and regimen (409, 412). Along with the genotype and associated polymorphisms, the enzymes metabolize other drugs too, and thus the effectiveness of tamoxifen is dependent on the intake of tamoxifen and any other drug taken simultaneously, for example paroxetine (406). In a separate study, it was noted that upregulation of the CYP3A4 enzyme played a crucial role in tamoxifen resistant BC cells and this was attributed partially to the 11,12-epoxyeicosatrienoic (11-12-EET) acid pathway and thus drugs targeting the CYP3A4/11,12-EET pathway may serve as suitable therapeutic drugs (413). A phase I trial conducted using Z-endoxifen as a treatment of choice rather than tamoxifen was performed to bypass the metabolism of the drug and thus differential effects due to the CYP and other enzymes, showed promising anti-tumor effects unaffected by toxicity (414).

## 9. Immune System

The complicated crosstalk between the cancer cells and immune cells at the site of the tumor has led researchers to harness the unique capability of the immune system to destroy and at times shrink the size of the tumor. In BC as in other cancers, increased levels of immune cells and soluble mediators like cytokines and some chemokines, predict poor prognosis. It has been reported that tumor associated macrophages (TAM) are associated with low survival rates and that the TAMs exhibit features that promote angiogenesis, migration, EMT and metastasis by suppressing anti-cancer immunity (415, 416). Interestingly, the levels of interleulin-1β (IL-1β) and tumor-necrosis factor α (TNFα) are higher in metastatic ER+ BC patients, where these cytokines activate the NF-κB and lead to endocrine resistance (417, 418). Antibodies and molecules directed against transcription factors upstream of NF-κB in the NF-κB pathway has been observed to restore sensitivity in cell line models of endocrine resistant BC (419, 420). IL-1β dependent activation of the ER, due to phosphorylation at the serine 305 locus of the protein by IKKβ leads to growth and proliferation of BC cells in the absence of E2, thus providing evidence for a prominent role for cytokines in BC (421).

Although in BC it is the negligible immunogenicity of the tumors that limits the effectiveness of immunotherapies like checkpoint inhibitors, yet the immune system plays an essential role in the development and branching of ductal and luminal epithelial differentiation (422, 423). According to some researchers, the immune system appears to play a very prominent role in the triple negative BC setting (ER-negative, PR-negative, and HER2-negative), and has a limited role in the endocrine sensitive setting (ER+ BC) (424, 425).

Thus, although there is a role of both the innate and adaptive immune cells in BC and immune mechanisms have been more intensely studied in ER negative patients, recent studies have focused on studies suggesting a role for the adaptive arm of the immune system in ER+ patients, namely the TILs (22). In post-menopausal women with ER + BC receiving neoadjuvant AI, anastrozole, an inflammatory gene expression signature was observed in baseline samples (426). The cluster of genes expressed were suggestive of infiltrating immune cells and these are associated with poor response to endocrine therapy. The most recent study focused on identifying a prognostic value for immune scores and associating it to the relapse in ER + BC patients receiving neoadjuvant AI, anastrozole or tamoxifen (23). The study reveals the importance of spatial heterogeneity of TILs and a crucial role for immune memory of tumor immune cells in ER + patients.

In the ER+, PR+, and HER2+ setting, it is the therapy based on monoclonal antibodies against HER2 that activate the antibody-dependent cytotoxic killing ability of natural killer (NK) cells through the adaptive arm of the immune system (427). hTERT is also capable of eliciting an immune response and it has been reported that there was an abundance of hTERT peptide 1540 specific CD8+ T cells in some cancers and in the metastatic BC setting, patients immunized with the peptide presented with increased TILs and necrosis of some tumorigenic areas (428).

Checkpoint inhibitors, anti-CTLA4 and anti-PD1 have been reported to elicit anti-cancer responses, although studies in the ER+ luminal BC subtype, demonstrate that due to the low titer of TILs in these patients, it is not the therapy of choice (422). In a study aimed at tumor rejection in HER+ metastatic BC patients, trastuzumab was given in combination with anti-PD1 (PANACEA study) and it was reported that the objective response rate in the PDL1+ BC patients was 15.2% and 0% in the PDL1-negative arm of the study (422). The myeloid derived suppressor cells (MDSCs) are another set of myeloid derived cells that have revealed their importance in promoting resistance to therapies (429). Therapies involving the immune system hold promise for future efforts to overcome BC endocrine resistance.

## 10. miRNA and Extracellular Vesicles

MicroRNAs (miRNA's) are small (~22-nucleotide) regulatory non-coding RNA molecules and miRNA deregulation was first reported in 2005 in BC (430). Investigators have reported that miRNAs can regulate the network/cascade of signal transduction pathways associated with endocrine resistance by several mechanisms: upregulating drug efflux transporters and anti-apoptotic proteins, promoting EMT and forming cancer stem cells (431).

Aberrant expression of specific miRNAs has been implicated in the development of tamoxifen resistance. Gene expression profiling has identified differential expression of miRNA expression profiles between tamoxifen resistant and sensitive BC cell lines (431–433). A miRNA library recently identified a plethora of miRNAs involved in TAM sensitivity in MCF7 cells (434). A previous study demonstrated that overexpression of miR-101, led to TAM resistance in MCF7 cells grown in an E2 free medium, mediated by the activation of AKT (435). The miR-519a regulates tumor suppressor genes within the PI3K and cell cycle proteins conferred endocrine resistance (436). The miR-451a, promotes endocrine resistance through a reduction in autophagosomes (437). Basically, The PI3K/AKT/mTOR pathway plays a crucial role in metastasis and endocrine resistance and overexpression of miR-451a enhances sensitivity to tamoxifen by repressing 14-3-3ζ expression along with reduced p-AKT and p-mTOR and an increase in the ERα expression. miR221/222 targets p27/Kip1, which is a cell cycle inhibitor in TAMR BC cells and promotes proliferation of cells even in the absence of E2 (432). Interestingly, expressing miR-320a in TAMR BC cells, leads to re-sensitization through downregulation of the c-MYC and CYCLIN D1 (438). Again, an increase of miR-873 in TAMR BC cells led to a restoration in the tamoxifen sensitivity through CD3 (439). Another study, highlighted the importance of the miRNA-375, in TAMR BC cells, where re-expression of miR-375, led to a reversal in tamoxifen resistance and associated EMT like behavior in these cells (440). BC stem cells also activate the HIF1α pathway during hypoxic conditions (441).

Extracellular vesicles (EVs) derived from cancer cells are similar to lipid vesicles, and contain oncogenic material in the form of overexpressed oncogenic genes/proteins/mediators/nucleic acids/non-coding RNA's and metabolic enzymes. They typically home into specific recipient cells where they can trigger a separate cascade of molecules and associated pathological response (442, 443). An analysis of the contents within the EV could shed light on their role in endocrine resistance and metastasis. EVs have been implicated in transmitting tamoxifen resistance from the TAM resistant cells to the TAM sensitive cells via the EVs containing miR-221/222 (434). Cancer biomarkers identified include HER2 and HLA-G, which is associated with circulating tumor cells and promotes proliferation, therapy resistance and metastasis (444). The translational capacity of miRNAs and EVs hold promise toward identifying therapies to combat endocrine resistance.

## Conclusion

The molecular mechanisms underlying resistance to endocrine therapy have been much studied and these studies have provided novel insights and yielded potentially new therapeutic strategies that may overcome endocrine resistance in BC. While an improvement in the quality of life and survival of women with BC has increased with the discovery of novel ER targeted therapies, endocrine resistance still remains at large. The ER signaling pathway represents a complex cascade of events with several regulators and comprehensive crosstalk with and between pathways, thereby resulting in the emergence of endocrine resistance. The authors view stem cells and the immune system as a focus toward all future studies on overcoming endocrine resistance in BC and other solid cancers. The soluble microenvironment around the BC, which consists of hormones, growth factors, cytokines, ROS, and various other types of metabolic compounds determine the effectiveness of the immune system in eliminating or shrinking the size of tumors. The upcoming efforts should now be to study potential biomarkers for definitive use in the clinic in greater detail. It is well-understood that results that appear promising in cell lines will not entirely translate into clinically reproducible results, thereby rendering clinical validation as a necessary step in evaluating novel therapeutic strategies. Advances in integrative approaches examining the transcriptomic and proteomic profiles in combination with robust bioinformatics support studying the differences between normal and resistant tumor tissue from a large dataset of patients will help tease out the differences/similarities between the two datasets and furthering our understanding. A focused study of the immune cell infiltrates and tumor spatial architecture around the site of the cancer, polymorphisms within metabolic enzymes and oxidative stress milieu are also aspects that will help clinicians in treatment decisions. This approach will help identify novel mediators, molecules and pathways of interest thereby resulting in tailor-made treatment for different sets of patients with similar clinical symptoms and molecular profiles.

## Author Contributions

All authors contributed to the text and design of the review and approved the final manuscript.

### Conflict of Interest Statement

JS sat on SABs for Celltrion, Singapore Biotech, Vor Biopharma, TLC Biopharmaceuticals and Benevolent AI, has consulted with Lansdowne partners, Vitruvian and Social Impact Capital and he Chairs the Board of Directors for BB Biotech Healthcare Trust and Xerion Healthcare. The remaining authors declare that the research was conducted in the absence of any commercial or financial relationships that could be construed as a potential conflict of interest.
